# The role of water in the laboratory thermal advancement of immature type I kerogen from the Cretaceous Qingshankou Formation in China

**DOI:** 10.1038/s41598-023-38013-z

**Published:** 2023-07-04

**Authors:** Majid Safaei-Farouji, Thomas Gentzis, Bo Liu, Zhongliang Ma, Liu Wang, Yaohui Xu, Mehdi Ostadhassan

**Affiliations:** 1 Department for Applied Geosciences and Geophysics, Montanuniversitaet Leoben, 8700 Leoben, Austria; 2 Core Laboratories, Reservoir Geology Group, 6316 Windfern Road, Houston, TX 77040 USA; 3 Hubei Key Laboratory of Petroleum Geochemistry and Environment, Yangtze University, Wuhan, 430100 China; 4 Key Laboratory of Continental Shale Accumulation and Efficient Development, Ministry of Education, Northeast Petroleum University, Daqing, 163318 China; 5 Wuxi Research Institute of Petroleum Geology, Sinopec Petroleum Exploration & Production Research Institute, Wuxi, 214126 China; 6 Institute of Geosciences, Marine and Land Geomechanics and Geotectonics, Christian-Albrechts- Universität, 24118 Kiel, Germany

**Keywords:** Geochemistry, Coal

## Abstract

To understand variations in geochemistry, organic petrology, and chemical composition of crude oil and byproducts, an immature sample from the Cretaceous Qingshankou Formation in the Songliao Basin, China, was analyzed by anhydrous and hydrous pyrolysis (AHP/HP) at a wide range of temperatures ranging from 300 °C to 450 °C. The geochemical parameters: TOC, S_2_, HI, and T_max_ obtained from Rock–Eval pyrolysis showed both a decrease and an increase as thermal maturity progressed under HP and AHP conditions. Gas chromatography (GC) analysis showed the presence of n-alkanes in the C_14_ to C_36_ range in both expelled and residual byproducts, a Delta-shaped configuration although many samples had a gradually reducing (tapering) trend toward the high range. Gas chromatography-mass spectrometry (GC–MS) analysis revealed both an increase and a decrease in biomarker and very small changes in aromatic compound variations with increasing temperature during pyrolysis. To be more specific, C_29_T_s_ biomarker increased with temperature for the expelled byproduct, while the opposite trend was observed for the residual one. Next, The T_s_/T_m_ ratio initially increased and then decreased with temperature while the C_29_H/C_30_H ratio fluctuated for the expelled byproduct but increased for the residual. Moreover, the GI and C_30_ rearranged hopane to C_30_ hopane ratio remained unchanged whereas the C_23_ tricyclic terpane/C_24_ tetracyclic terpane ratio and the C_23_/C_24_ tricyclic terpane ratio showed varying trends with maturity alike the C_19_/C_23_ and C_20_/C_23_ tricyclic terpane. Ultimately, based on organic petrography observations, increasing the temperature resulted in higher bitumen reflectance (%Bro, r) and optical and structural alterations in the macerals. The findings of this study provide valuable insights for future exploration endeavors in the studied region. Moreover, they contribute to our understanding of the significant role of water in the generation and expulsion of petroleum and associated byproducts, thereby facilitating the development of updated models in this field.

## Introduction

To replicate the natural process of organic matter thermal advancement in the subsurface and to investigate what occurs to macerals after they are buried, two kinds of pyrolysis methods are used in the laboratory: anhydrous and hydrous pyrolysis (AHP/HP). AHP is distinguished by the lack of water in the reactor during artificial maturation, which is the primary difference from HP. This means that in HP, liquid water, not H_2_O vapor or supercritical fluid, is used to come in contact with the heated sample^1^. Anhydrous or dry pyrolysis has been widely used to simulate the natural pathway of thermal progression in a laboratory setting over the geological time scale^2–4^. Additionally, water has been proposed to be critical for both natural and experimental oil generation since it can supply hydrogen, which is required to produce oil from organic matter. Water also plays an important role in the evolution of the expelled oil and gas. Without water flowing in the pyrolysis chamber, hydrocarbon-enriched oil will be difficult to separate from polar-enriched bitumen, and there will be insufficient volume change to extract the oil from the bitumen-soaked rock^5^.

Temperature stress triggers the bitumen's covalent bonds to break, leading to the emergence of a hydrocarbon-rich oil. The produced oil is discharged from the rock due to its immiscibility with the water-saturated bitumen and the subsequent net volume increase^6–8^. When the thermal maturation of organic matter is simulated using hydrous pyrolysis, the entire process proceeds under the presumption that oil is expelled as a result of its formation within the rock, as initially postulated by Momper^9^. Several studies^7,8,10–12^ have proposed that the HP process better follows the natural maturation process than closed and open system AHP. Thus, HP should be considered as the most appropriate pyrolysis approach for artificially simulating the process of petroleum generation and expulsion^5^. Moreover, it was argued that the HP approach yields more realistic activation energies for the entire processes that will result in the formation of hydrocarbons^8^.

Both AHP and HP have been carried out to investigate and compare the impact of water on the maturation of organic matter. For instance, Lewan and Roy^13^ conducted both AHP and HP experiments on a sample with Type I kerogen from the Mahogany oil shale (Eocene Green River Formation) in order to determine the effect of water on hydrocarbon formation. Water played a more important role in transforming bitumen to oil at 350 °C than transforming kerogen to bitumen at 330 °C. Additionally, the authors observed fractures in the sample subjected to HP, but no such fractures were identified in the sample heated under AHP. They hypothesized that the emergence of these fractures in the hydrous recovered sample but not in the anhydrous one, would signify a physiochemical role that water plays during the reactions.

Additionally, Spigolon et al.^5^ used HP to investigate the content and level of petroleum created as thermal maturity evolved in a Brazilian lacustrine source rock of Type I kerogen. The API gravity, C_15_ + percentages, and gas/oil ratios of the HP test were equivalent to those of natural hydrocarbons obtained from the same source rock. The alkane compositions of the HP expelled oils were found to be comparable to crude oil that was produced naturally from a similar Lower Cretaceous Brazilian lacustrine source rock with Type I kerogen. By comparing the composition of hydrocarbons derived from these lacustrine source rocks between natural and hydrous pyrolysis, the authors validated that HP better characterizes petroleum systems and the influences of maturation and expulsion on hydrocarbon composition and other attributes.

Although the focus of the present study is on lacustrine kerogen Type I, most published HP/AHP studies have been conducted on the more commonly found kerogen Types II and III. For example, Lewan^7^ demonstrated that pyrolysis of Type II kerogen in the Woodford Shale produced higher liquid petroleum yields in the presence of water than in the absence of water. Macerals experienced extensive alterations during the thermal maturity phase, including chemical to morphological and structural deformations^14^. The above authors described the change in the color and reflectance of vitrinite with increased temperature under HP conditions and observed that the heating process caused vitrinite to devolatilize. They also stated that as the temperature increased, the pores became more frequent and the fractures became dominant. Liptinite macerals attained higher reflectance and a weaker fluorescence when they were heated. As a result, the above studies can be used for comparison purposes with the current study.

Given the significance of studying the geochemical evolution of organic matter (OM) of various biogenic origins and depositional environments that will result in a variation in the chemistry of the liquid petroleum products, we explored the morphological and structural alterations of macerals in the Cretaceous Qingshankou Formation, Songliao Basin, China, under both AHP and HP scenarios over a wide temperature range. The primary objective of the study is to determine the effect of water on the macerals during the process to support proposing an updated model for the role of water in petroleum expulsion and the chemistry of the yielded products. In addition, changes in geochemical parameters acquired from programmed pyrolysis (Rock–Eval 6), gas chromatography (GC), and GCMS (GC-Mass Spectrometry) were also investigated in order to provide a more comprehensive view of the maturation routes, which is expected to be controlled by the biogenic origin of the OM. Although there are several publications examining the Qingshankou Formation from an organic geochemistry point of view^15–18^, to the best of our knowledge there is not a single study that has assessed the role of water in the maturation of the Qingshankou Formation from many different perspectives. The results of this study will enable researchers who are studying productive intervals in this complex basin to successfully carry out oil-oil correlations as well as the oil-source correlations. This is in addition to the fact that the large temperature program that has been used here, from immature to post-gas generation window, has presented a unique opportunity to understand changes in the maceral morphology, reflectance and fluorescence, as well as to characterize the hydrocarbon products generated from Type I kerogen.

## Geology

**Figure 1:**
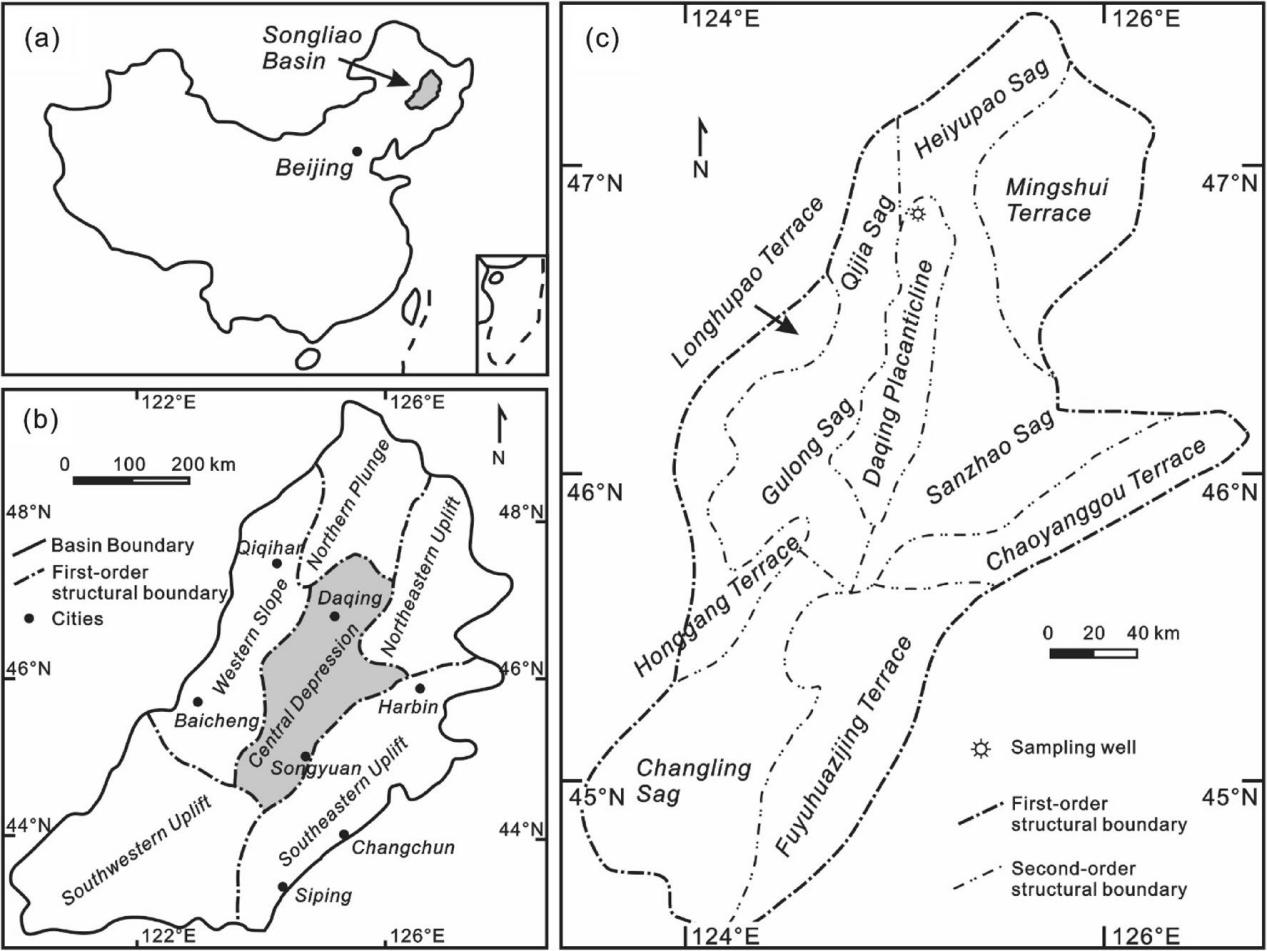
The location map of the Songliao Basin (modified from Liu et al.^21^).

**Figure 2:**
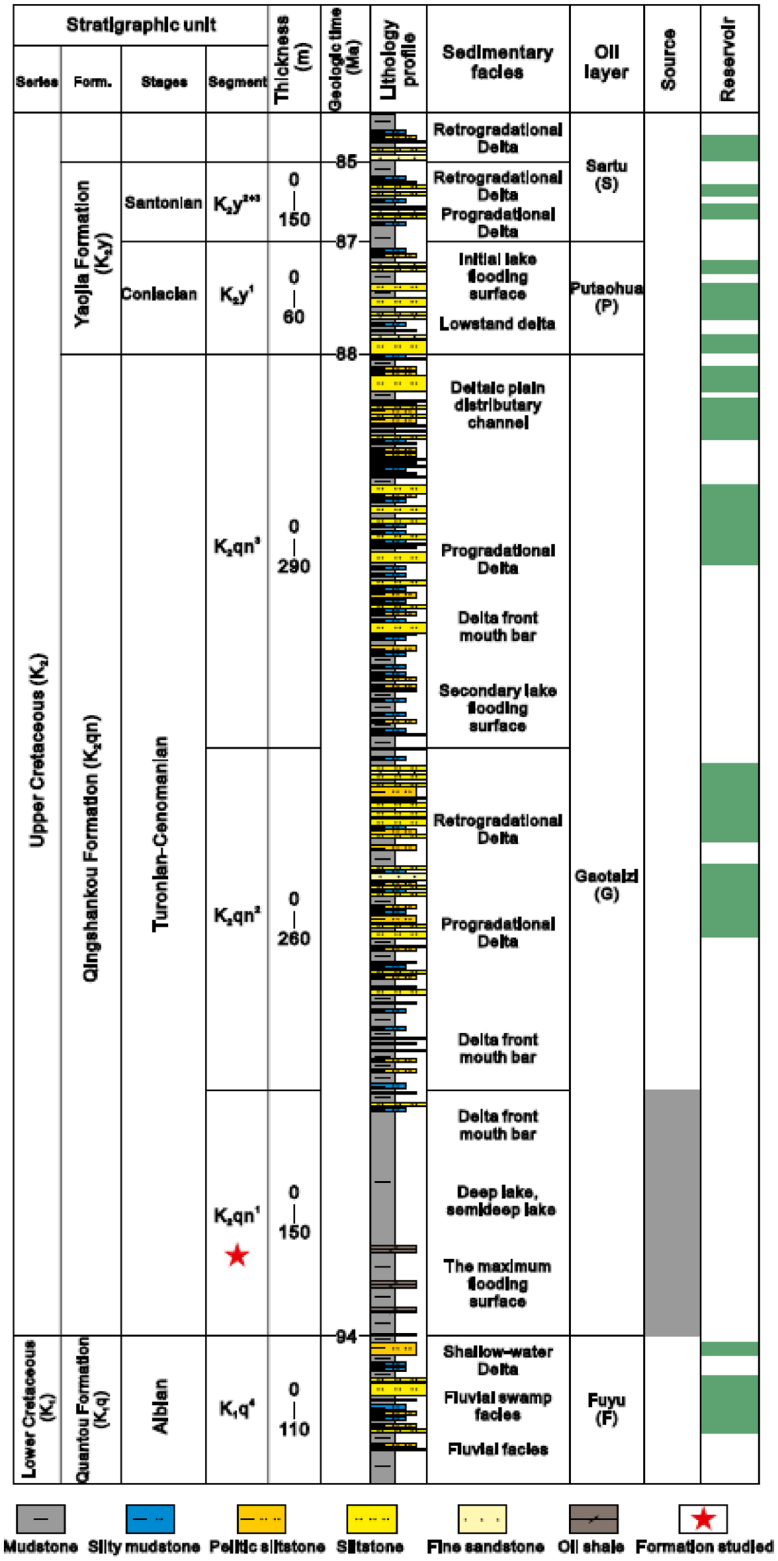
Stratigraphic column of the Qingshankou Formation and its members in the study area (modified from Liu et al.^21^).

The Songliao Basin (Fig. 1), with a covering area of 260,000 km^2^, is one of the world’s largest continental Cretaceous basins containing lacustrine rocks. The basin is an intracontinental non-marine basin and hosts rich conventional and unconventional hydrocarbon resources. It also contains the giant Daqing oil field in its center, which is the largest known oil field in China in terms of production^19^. Since it was discovered in 1960, the Daqing oil field has produced more than 50 million tonnes of crude oil annually for 27 consecutive years. In recent years, its annual oil and gas production has stood above 40 million tonnes of oil equivalent. Peak production reached 800,000 barrels of oil per day in 2021. Current production (2023) of oil is 600,000 barrels per year (Wikipedia.com; accessed in May 2023). The Cretaceous-age Qingshankou Formation, which is a major oil producing shale formation, was deposited in a relatively deep lake environment impacted by frequent marine incursions during a time of global sea-level rise^20^. Generally, the Qingshankou Formation consists of three members, known as K_2_qn^1^, K_2_qn^2^, and K_2_qn^3^ (Fig. 2) that vary in their lithology^21^. The K_2_qn^1^ comprises of dark-grey mudstone and shale interbedded with sequences of coarse to fine sandstone^22^. In comparison to the top member’s assemblage of white grey sandstone (K_2_qn^3^), the K_2_qn^2^ member, from where the sample was retrieved, is composed of grey-green mudstone/shale and white–grey fine sandstone and siltstone^23^. General details about this formation can be found in Liu et al.^21^.

## Materials and methods

### Anhydrous pyrolysis (AHP) and hydrous pyrolysis (HP)

Experiments of AHP and HP were conducted in a 1-L Hastelloy C-276 reactors (Parr Instrument Co.). The water-to-rock ratio was determined using steam tables and bulk rock densities based on Lewan^1^. This was performed to ensure that the rock sample remained completely submerged in liquid water throughout the pyrolysis process. Without extracting or drying the samples, they were crushed to gravel size (0.5–2.0 cm). In each experiment, the reactor was loaded with 500 g of shale material. The reactor was switched off and allowed to vent for a few minutes. After the reactors were loaded, they were sealed with a stainless steel 316 gasket and an eight-bolt split-ring head. Helium was subsequently fed into the reactors to a pressure of 7 MPa, to detect possible leaks using an electronic leak detector. Helium pressure in the reactors was reduced to between 2.3 and 2.5 kPa, and its pressures and temperatures were monitored. Finally, over the course of the experiment, the gas from the reactor headspace was gradually drained into a system with a known volume^24^. After each AHP and HP test, byproducts were collected for further petrological, geochemical, and compositional study. The temperatures used in the pyrolysis experiments were 300 °C, 325 °C, 350 °C, 365 °C, 400 °C and 450 °C. The above temperature range was selected because it is the one typically used to study the effects of AHP and HP on kerogen. It is important to know that water becomes supercritical at 373 °C and 7376 kPa. Since the pressure in the reactor was lower, the water never reached a supercritical (vapor) condition.

### Open-system programmed (Rock–Eval) pyrolysis

To collect geochemical data from the specimens following each stage of AHP/HP, programmed pyrolysis following the Basic/Bulk-Rock method was performed with a Rock–Eval 6 (RE-6) apparatus^25^. To do so, 60 mg of powdered bulk rock was placed in crucibles for three minutes in an inert (nitrogen) environment and then heated to 650 °C at a rate of 25 °C/min. The residual organic carbon from the pyrolysis stage was subsequently burned in a second oven (oxidation oven). This procedure will yield TOC (weight percent), which is the sample's organic richness, S_1_ and S_2_ (mg HC/g Rock), the sample’s free oil content and the remaining hydrocarbon potential, respectively, and the T_max_ (°C), which represents the sample’s thermal maturity. Other indices such as HI (S_2_ $\times$ 100/TOC), OI (S_3_ $\times$ 100/TOC), and S_1_ + S_2_ will be obtained using these parameters. Behar et al.^25^ provided a detailed description of the pyrolysis technique and how to calculate relevant geochemical parameters.

### Organic petrography

To perform organic petrography and reflectance (%R_O_) measurements, all samples were processed as polished whole-rock blocks (pellets). Samples were crushed to an average particle size of 840 μm and then pelletized using an epoxy resin (Epo-Thin^TM^) and hardener and allowed to firm for 8 h. Next, the pelletss were polished with 600 and 400 grit polishing cloth sets, accompanied by an automated Buehler EcoMet/AutoMet 250 system using 0.3 and 0.05 μm alumina powder to achieve the desired roughness and a relief-free surface. We investigated the random reflectance of bitumen particles due to the shortage or absence of vitrinite in the examined samples and to be consistent throughout the entire process. Each sample was subjected to fifty (50) BR_O_ (bitumen reflectance) measurements. The Ro measurements were taken using a Zeiss Axio Imager A2m microscope, glass standards that has %Ro values of 1.0, 1.36, and 1.78, and software licensed by CRAIC Technologies. All Ro measurements are random (%Ro, r).

### OM extraction, GC, and GCMS analysis

Bitumen extraction was carried out on powdered samples (30–40 gr each) over a 72-h period using a Soxhlet extractor and an azeotropic mixture of dichloromethane (DCM) and methanol (CH_3_OH) (93:7 vol: vol). Additionally, sulfur was removed using activated copper, and n-hexane was used to de-asphalt the recovered bitumen. The saturate portion of extracted bitumen was dissolved in hexane and analyzed using a gas chromatography column equipped with the matching standards and experimental procedures as follows: the temperature was ramped up to 300 °C at a rate of 4 °C/min in a Chrompack CP-9000 equipped with a glass capillary column (25 m 0.25 mm i.d.) coated with DB-5, and then maintained at 300 °C for 30 min. The biomarkers were separated using a Thermoquest 2000 gas chromatograph. The following oven temperature program was held at 60 °C for 2 min, increased by 3 °C/min to 280 °C, and maintained for 40 min. At 280 °C, the segregated molecules were transferred to a mass spectrometer (MS). Thermo Finnigan selected ion monitoring (SIM) was used to determine steranes (m/z = 217) and triterpanes (m/z = 191).

## Results

### Rock–Eval pyrolysis

The results of Rock–Eval pyrolysis parameters for both AHP and HP conditions and for both un-extracted and extracted organic matter are summarized in Table 1. Considerable variations in terms of both decreasing and increasing trends occurred as the temperature increased in both HP and AHP as well as for un-extracted and extracted OM.

**Table 1:** Rock–Eval derived parameters with increasing temperature under AHP and HP conditions for the un-extracted and extracted samples.

Condition	Un-extracted							Extracted						
	TOC	Tmax°C	S_1_	S_2_	S_3_	HI	OI	TOC	Tmax°C	S_1_	S_2_	S_3_	HI	OI
Original	2.28	441	0.45	15.51	0.20	680	8	2.23	444	0.13	13.28	0.49	595	21
HP-300	2.27	447	0.18	13.31	0.20	586	8	1.98	441	0.11	9.46	0.51	477	25
HP-325	2.08	444	0.66	11.58	0.17	556	8	1.63	441	0.16	4.42	0.46	271	28
HP-350	2.06	443	1.99	7.77	0.14	377	6	1.43	440	0.13	1.71	0.39	119	27
HP-365	1.97	439	3.22	4.86	0.12	246	6	1.30	455	0.06	0.18	0.30	13	23
HP-400	1.35	450	0.53	0.40	0.15	29	11	1.58	455	0.06	0.01	0.31	0.63	19
HP-450	1.56	454	0.20	0.05	0.13	3	8	2.30	441	0.07	11.82	0.26	513	11
AHP-300	2.32	446	0.26	13.34	0.16	575	6	2.27	446	0.10	12.41	0.40	546	17
AHP-325	2.21	443	0.38	12.10	0.14	547	6	2.09	446	0.08	9.38	0.50	448	23
AHP-350	1.90	441	0.72	7.87	0.23	414	12	1.39	440	0.05	3.00	0.49	215	35
AHP-365	1.58	436	0.81	3.70	0.10	234	6	1.21	446	0.05	0.82	0.21	67	17
AHP-400	1.17	558	0.08	0.17	0.11	14	9	1.12	455	0.06	0.12	0.18	10	16
AHP-450	0.90	607	0.13	1.66	0.15	184	16	1.05	418	0.19	2.26	0.22	215	20

**Figure 3:**
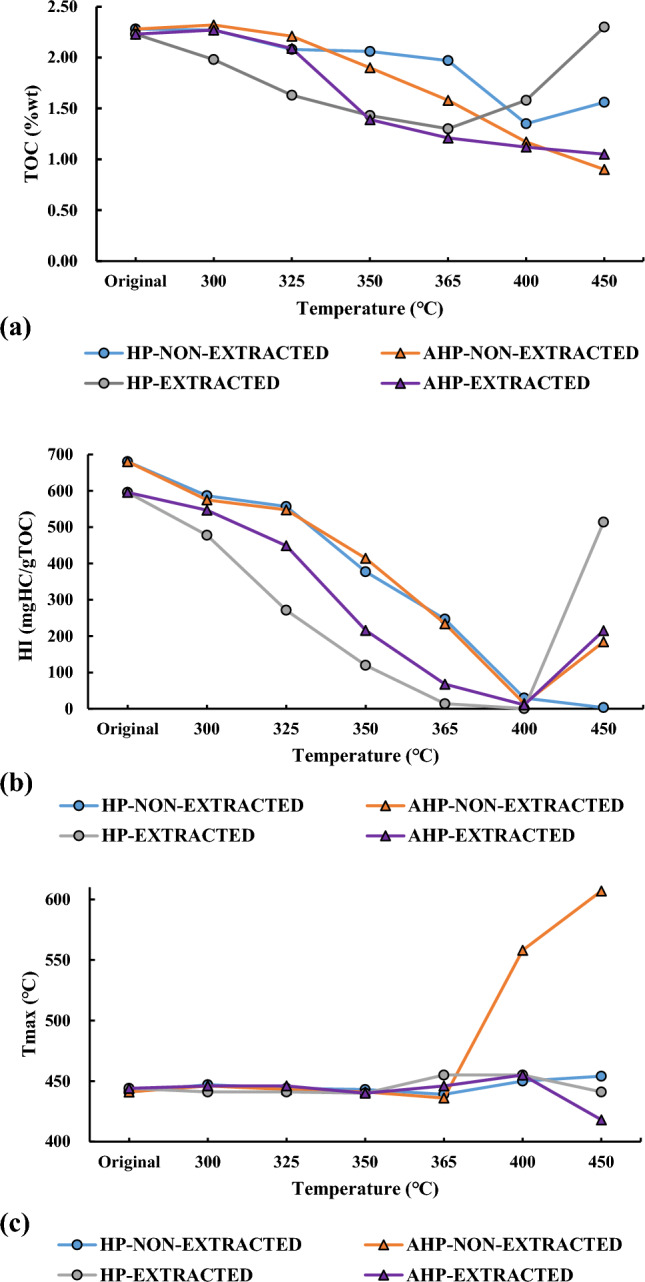
Changes in (**a**) TOC (**b**) HI and (**c**) T_max_ vs. temperature during AHP and HP tests for both un-extracted and extracted OM.

Figure 3a shows that under AHP conditions, there is a constant decrease in TOC as temperature increased for both un-extracted and extracted OM. Considering the AHP test, the TOC of the un-extracted and original (unheated) OM decreased from 2.28 wt% to 0.9 wt% when the temperature reached 450 °C. Likewise, in the case of extracted OM, the TOC of the immature sample decreased from 2.23 wt% to 1.05 wt% when the temperature reached 450 °C. This translates to approximately a 40% reduction in the un-extracted sample and a nearly 53% reduction in the extracted OM during the AHP test. A similar trend can be observed for the original sample in the presence of water (HP test) when the un-extracted OM temperature increased from 300 °C to 400 °C. In this interval, TOC decreased from 2.28 wt% to 1.35 wt% and then increased slightly to 1.56 wt% when the temperature reached 450 °C. Regarding the extracted OM, TOC of the unheated OM decreased from 2.23 wt% to 1.30 wt% between 300 °C and 365 °C and then increased to 2.30 wt% at 450 °C (Fig. 3a).

The HI value of the original (unheated) sample decreased significantly under both AHP and HP conditions and for both un-extracted and extracted OM (Table 1, Fig. 3b). Therefore, the presence or absence of water did not have a notable impact on decreasing the HI as pyrolysis temperature increased. However, this decreasing trend is more notable between 300 °C and 400 °C during the HP test when the HI value of the unheated and un-extracted OM decreased drastically from 680 (mg HC/g TOC) to 3 (mg HC/g TOC), a 99.5% decrease. In the presence of water, the HI value of the extracted OM was reduced from 595 (mg HC/g TOC) to 0.6 (mg HC/g TOC) between 300 °C and 400 °C, nearly a 99.8% reduction. However, the HI value reached 513 (mg HC/g TOC) when the temperature increased to 450 °C.

Regarding the AHP test, a notable decrease in HI was observed when the un-extracted OM was heated between 300 °C and 400 °C (Table 1, Fig. 3b). In this interval, the HI decreased from 680 (mg HC/g TOC) to 14 (mg HC/g TOC), approximately a 98% decrease. When the temperature increased from 400 °C to 450 °C, the HI value increased from 14 (mg HC/g TOC) to 184 (mg HC/g TOC). In the case of the extracted OM (Table 1, Fig. 3b), the HI decreased from 595 (mg HC/g TOC) to 10 (mg HC/g TOC) a nearly 98% decrease. Thus, both un-extracted and extracted OM experienced a similar amount of reduction in HI in the AHP test and in the temperature interval of 300 °C–400 °C.

Regarding changes in the T_max_ values, there is a similar trend for the un-extracted OM under both HP and AHP conditions. The T_max_ values of the original sample increased at 300 °C and decreased up to 365 °C. Then, the T_max_ increased until the final temperature of the HP test (450 °C) (Table 1, Fig. 3c). A similar increasing trend can also be observed during the AHP test. However, it is important to note that there was a general increase in T_max_ for the un-extracted OM in HP and AHP settings. The T_max_ value of the original OM is 441 °C and reached 454 °C at the final temperature of 450 °C under HP conditions. Similarly, during the AHP test, the T_max_ value of the original OM increased to 607 °C at 450 °C (Table 1, Fig. 3c). Thus, a higher increase in T_max_ values was noted under AHP conditions than under HP. Regarding changes in the T_max_ values of the extracted OM, irregular fluctuations were noticed when the temperature increased in both HP and AHP tests (Table 1, Fig. 3c).

### Gas chromatography (GC)

Gas chromatograms (see Supplementary Data, Figs. A and B) and corresponding calculated parameters for both expelled and residual byproducts (Tables 2 and 3) were used to understand the variations in normal alkanes and isoprenoids with increasing temperature during AHP and HP experiments.

**Table 2:** GC results of the expelled byproduct under HP and AHP conditions.

Sample/Condition	Pr/nC_17_	Ph/nC_18_	Pr/Ph	C_13_	C_14_	C_15_	C_16_	C_17_	C_18_	C_19_	C_20_	C_21_	C_22_	C_23_	C_24_	C_25_	C_26_	C_27_	C_28_	C_29_	C_30_	C_31_	C_32_	C_33_	C_34_	C_35_	C_36_
HP-300 °C	0.61	0.34	0.19	–	–	–	–	0.20	1.88	6.31	10.70	14.01	15.07	14.48	10.29	8.74	5.26	4.20	2.61	2.15	1.11	0.96	0.52	0.35	0.21	0.11	–
HP-325 °C	0.57	0.31	0.42	–	–	–	0.09	0.77	3.27	7.10	10.42	13.24	14.29	13.73	9.94	8.76	5.31	4.31	2.59	2	1.05	0.81	0.43	0.25	0.09	-	–
HP-350 °C	0.3	0.17	0.43	–	–	–	0.08	0.8	3.19	6.88	10.06	12.56	13.42	12.85	9.89	9	5.95	4.88	3.17	2.44	1.43	1.06	0.63	0.47	0.25	0.13	–
HP-365 °C	0.31	0.11	0.36	–	–	–	-	0.07	0.59	3.47	8.06	12.51	14.02	13.94	11.11	10.15	7.12	6.22	4.49	3.66	2.27	1.71	0.46	–	–	–	–
HP-400 °C	0.31	0.13	1.21	–	–	–	0.66	2.77	5.21	7.74	9.67	11.68	12.15	11.73	8.99	8.06	5.57	4.85	3.28	2.57	1.54	1.32	0.55	–	–	–	–
HP-450 °C	0.11	0.08	1.22	–	0.73	3.48	6.81	8.58	9.27	9.24	9.01	9.18	8.66	8.03	6.16	5.43	3.76	3.07	2.07	1.64	0.99	0.76	0.5	0.35	0.19	0.13	0.07
AHP-300 °C	0.51	0.39	0.66	–	–	–	0.71	4.55	8.95	12.36	13.94	14.34	12.79	10.12	5.97	4.26	2.18	154	0.86	0.58	0.32	0.30	0.15	0.10	0.05	–	
AHP-325 °C	0.41	0.28	0.47	–	–	–	0.28	2.58	7.85	12.01	13.18	13.53	12.53	10.90	7.25	5.92	3.37	2.62	1.55	1.22	0.62	0.54	0.28	0.20	0.11	0.06	
AHP-350 °C	0.21	0.13	1.58	–	0.56	4.26	9.96	13.24	12.88	11.47	9.71	8.64	7.18	5.66	3.69	2.91	1.72	1.31	0.81	0.53	0.32	0.24	0.15	0.09	0.06	–	
AHP-365 °C	0.10	0.08	1.35	–	1.11	5.71	10.14	11.65	11.10	10.01	9.01	8.42	7.48	6.41	4.66	3.84	2.51	2.01	1.29	0.94	0.49	0.42	0.27	0.17	0.13	0.07	
AHP-400 °C	0.02	0.02	1.81	–	0.93	6.97	16.38	19.01	16.14	12.30	8.76	6.38	4.41	2.94	1.79	1.19	0.71	0.47	0.28	0.18	0.10	0.08	0.03	–	–	–	
AHP-450 °C	0.29	0.12	0.76	–	–	–	0.03	0.11	0.31	2.14	8.64	13.85	14.13	11.31	8.37	7.21	6.41	6.15	4.48	6.03	2.53	4.36	1.22	1.47	0.54	0.36	0.2

**Table 3:** GC results of the residual byproduct under HP and AHP conditions.

Sample/Condition	Pr/nC_17_	Ph/nC_18_	Pr/Ph	C_13_	C_14_	C_15_	C_16_	C_17_	C_18_	C_19_	C_20_	C_21_	C_22_	C_23_	C_24_	C_25_	C_26_	C_27_	C_28_	C_29_	C_30_	C_31_	C_32_	C_33_	C_34_	C_35_	C_36_	C_37_
HP-300 °C	0.49	0.36	1.03	–	0.1	0.8	2.09	3.43	4.47	5.81	7.19	9.23	10.76	11.48	9.09	8.7	5.69	5.11	3.41	2.96	1.86	1.46	1	0.79	0.44	0.33	0.19	0.12
HP-325 °C	0.4	0.27	1.05	–	–	0.26	1.45	3.05	4.27	5.73	7.22	9.25	10.83	11.52	9.34	9.13	6.31	5.46	3.7	3.19	1.98	1.59	1.1	0.86	0.51	0.38	0.23	0.16
HP-350 °C	0.15	0.09	0.96	–	–	-	0.75	2.93	5.21	7.25	8.88	10.54	11.41	11.33	9.32	8.83	6.35	5.1	3.51	2.69	1.59	1.2	0.78	0.61	0.33	0.22	0.13	–
HP-365 °C	0.08	0.5	1.03	–	–	0.41	2.07	4.71	6.86	8.5	9.79	10.52	10.62	10.04	8.22	7.19	5.5	4.35	3.11	2.33	1.51	1.12	0.83	0.57	0.38	0.23	0.16	0.1
HP-400 °C	0.03	0.03	0.81	–	–	0.46	3.53	8.27	11.21	11.66	11.39	10.53	9.34	8.08	6.23	5.09	3.89	3.07	2.13	1.57	1	0.71	0.47	0.32	0.2	0.11	–	
HP-450 °C	0.2	0.23	0.18	–	–	–	0.11	0.45	2.13	4.06	5.46	5.74	6.38	8.53	9.53	11.29	9.75	9.35	7.2	6.32	4.22	3.31	2.10	1.47	0.85	0.54	0.33	0.22
AHP-300 °C	0.39	0.31	1.08	–	0.17	1.73	4	5.35	6.24	7.36	8.47	10.11	10.89	10.67	7.91	7.09	4.37	3.71	3.26	2.01	1.12	0.81	0.58	0.44	0.22	0.16	0.09	
AHP-325 °C	0.35	0.26	1.04	–	–	0.4	2.12	4.23	5.6	7	8.37	10.14	11.17	11.24	8.65	8.10	5.39	4.48	2.93	2.42	1.44	1.11	0.76	0.56	0.33	0.24	0.14	0.09
AHP-350 °C	0.17	0.12	1.08	–	0.03	0.79	2.81	5.03	6.69	8.05	9.19	10.42	10.82	10.40	8.19	7.38	5.13	4.13	2.80	2.15	1.32	0.99	0.66	0.51	0.31	0.2	0.12	0.08
AHP-365 °C	0.1	0.06	1.32	–	0.25	1.99	4.94	7.29	8.59	9.33	9.57	9.87	9.92	8.91	7.07	6.14	4.21	3.44	2.33	1.68	0.99	0.76	0.5	0.34	0.21	0.13	0.09	0.05
AHP-400 °C	0.03	0.02	1.22	–	0.78	5.59	11.79	14.04	13.77	12.12	9.89	8.36	6.70	5.18	3.56	2.59	1.66	1.20	0.73	0.46	0.27	0.17	0.1	0.05	–	–		
AHP-450 °C	0.17	0.18	0.65	–	–	–	0.78	5.56	8.35	7.68	7.6	8.21	8.67	8.82	8.06	7.79	6.45	5.94	4.51	3.78	2.26	1.61	0.65	0.36	0.22	0.13	–	–

The parameters attained were Pr/nC_17_, Ph/nC_18_, and Pr/Ph for both expelled and residual byproducts. The resulting chromatographs show irregular variations in the content of normal alkanes. The quantity of low molecular weight (LMW) alkanes increased as temperature increased under both HP and AHP conditions. Regarding the expelled byproduct, a very high molecular weight (HMW) alkane (C_36_) appeared only at a high temperature of 450 °C for both HP and AHP tests (Table 2, Supplementary Data Fig. A). On the contrary, heavier normal alkanes were present more frequently in the residual byproduct. With respect to the expelled byproduct, although there were fluctuations in the content of measured alkanes, generally and under both HP and AHP conditions, the < C_19_ n-alkanes increased as the temperature increased (Supplementary Data Fig. Aa–f and Table 2). In the HP test, C_14_ and C_15_ normal alkanes were detected only at a final temperature of 450 °C. However, these normal alkanes appeared at a lower temperature (350 °C) during the HP test. Additionally, a general decrease in C_19_-C_33_ n-alkanes was observed. An increase in the content of > C_33_ n-alkanes with increasing temperature during both HP and AHP tests was also noticed along with an increase in the content of LMW alkanes. This is important because the very high molecular weight alkane (C_36_) was merely detectable at a temperature of 450 °C during both HP and AHP experiments (Supplementary Data Fig. Aa–f and Table 2).

Regarding the residual byproduct, heavy normal alkanes such as C_36_ and C_37_ were more frequently present compared to the expelled byproduct (Table 3). According to Table 4 and Supplementary Data Fig. Ba–f, it is evident that the above heavy alkanes increased as temperature increased under HP conditions. The highest values of the C_36_ and C_37_ n-alkanes were detectable at 450 °C. Likewise, comparing the GC results of expelled and residual byproducts suggests that the low molecular weight n-alkane of C_15_ is more dominant in the residual byproduct. However, as was observed in the expelled byproduct, the < C_19_ n-alkanes generally increased as temperature increased during both HP and AHP tests. Furthermore, a general decline in > C_33_ n-alkanes was noticed. When the temperature increased from 400 °C to 450 °C, there was a significant increase in the content of > C_25_ n-alkanes during both HP and AHP tests.

**Table. 4:** Changes in biomarkers and aromatic compounds with temperature increase under HP and AHP for the expelled byproduct.

Condition	C_24_ TT	C_19_/C_23_ tri	C_20_/C_23_ tri	C_23_/C_24_ tri	C_23_t /C_24_ tet	Ts/ Tm	C_29_Ts	GI	C_30_H	C_30_RH	C_30_RH/ C_30_H	C_29_H/ C_30_H	MPR	MPI	MDR	DBT/P
HP-300 °C	1.32	0.04	0.31	1.85	2.28	0.83	3.02	0.17	18.08	0.75	0.04	0.7	0.68	0.68	0.53	–
HP-325 °C	0.87	0.10	0.44	1.93	2.52	0.75	3.22	0.18	19.03	0.73	0.03	0.64	1.95	1.31	1.53	0.02
HP-350 °C	0.94	0.12	0.40	1.96	2.19	0.65	2.82	0.18	17.97	0.70	0.03	0.7	0.97	0.82	0.90	0.02
HP-365 °C	1.28	0.06	0.31	1.77	1.63	0.53	3.09	0.20	17.47	0.61	0.03	0.07	0.89	0.65	–	–
HP-400 °C	0.66	0.16	0.53	2.62	2.62	0.56	3.13	0.18	18.96	0.60	0.03	0.65	2.20	1.35	1.42	0.01
HP-450 °C	0.74	–	–	2.22	1.98	0.73	3.38	0.18	19.58	0.61	0.03	0.61	1.30	0.94	1.74	0.04
AHP-300 °C	1.27	0.21	0.77	2.22	2.97	0.95	2.98	0.15	16.37	0.69	0.04	0.06	1.39	1.54	1.36	0.18
AHP-325 °C	1.18	0.15	0.56	2.31	3.09	0.86	3.16	0.18	15.90	0.67	0.04	0.07	1.17	1.20	1.54	0.09
AHP-350 °C	1.19	0.46	1.19	2.24	3.79	0.74	2.32	0.21	10.44	0.43	0.04	0.06	1.07	0.49	1.59	0.06
AHP-365 °C	0.44	0.28	0.80	1.74	1.86	1.12	2.80	0.15	19.43	1.71	0.08	0.6	1.23	0.62	2.26	0.05
AHP-400 °C	1.33	–	1.07	2.04	3.09	1.13	2.80	0.09	15.08	1.06	0.07	0.66	2.42	1.39	5.23	0.02
AHP-450 °C	0.72	–	0.1	1.35	1.91	1	3.05	0.16	18.25	1.42	0.07	0.06	2.19	2.46	–	–

### Biomarkers and aromatic compounds

**Figure 4:**
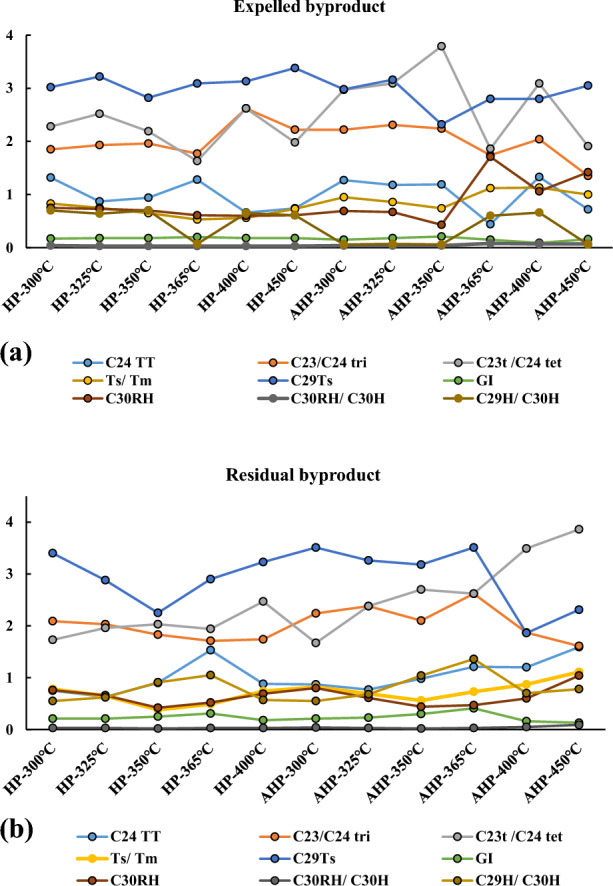
Changes in biomarkers values with increasing temperature under HP and AHP conditions for expelled (**a**) and residual (**b**) byproducts.

Diverse biological markers, including saturate and aromatic compounds were selected to investigate their variations with temperature advancement during HP and AHP experiments for both expelled and residual byproducts (Tables 4 and 5). Changes in biomarkers is presented in Fig. 4a for the expelled and Fig. 4b for the residual byproducts during AHP and HP tests. As it is evident, generally, biomarker values show fluctuations when they are subjected to heating during both AHP and HP tests. Interpretation of these variations will be presented in the Discussion section.

**Table 5:** Changes in biomarkers and aromatic compounds with temperature advancement under HP and AHP for the residual byproduct.

Condition	C_24_ TT	C_19_/C_23_ tri	C_20_/C_23_ tri	C_23_/C_24_ tri	C_23_t/C_24_ tet	Ts/Tm	C_29_Ts	GI	C_30_H	C_30_RH	C_30_RH/ C_30_H	C_29_H/C_30_H	MPR	MPI	MDR	DBT/P
HP-300 °C	0.75	0.13	0.58	2.09	1.73	0.78	3.40	0.21	19.81	0.76	0.03	0.55	1.08	0.41	1.28	0.04
HP-325 °C	0.63	0.14	0.53	2.03	1.96	0.65	2.88	0.21	19.62	0.66	0.03	0.62	1.39	0.45	1.42	0.04
HP-350 °C	0.9	0.26	0.56	1.83	2.03	0.38	2.25	0.25	17.14	0.42	0.02	0.91	1.01	0.50	1.24	0.04
HP-365 °C	1.53	0.52	0.93	1.71	1.94	0.49	2.90	0.31	14.10	0.52	0.03	1.05	1.09	0.55	2.18	0.05
HP-400 °C	0.88	0.11	0.33	1.74	2.47	0.74	3.23	0.18	18.61	0.69	0.03	0.57	1.65	0.99	2.32	0.04
HP-450 °C	–	–	–	–	–	–	–	–	–	–	–	–	–	–	–	–
AHP-300 °C	0.87	0.13	0.57	2.24	1.67	0.82	3.51	0.21	19.88	0.80	0.04	0.55	0.90	0.48	1.99	0.14
AHP-325 °C	0.77	0.17	0.64	2.38	2.38	0.69	3.26	0.23	17.91	0.61	0.03	0.68	0.89	0.52	1.49	0.06
AHP-350 °C	0.98	0.21	0.64	2.10	2.70	0.56	3.18	0.30	14.89	0.44	0.02	1.04	1.08	0.51	1.82	0.04
AHP-365 °C	1.21	0.30	0.89	2.62	2.62	0.73	3.51	0.41	12.24	0.47	0.03	1.36	1.29	0.64	2.28	0.04
AHP-400 °C	1.20	0.28	0.78	1.87	3.49	0.87	1.86	0.16	11.51	0.60	0.05	0.7	2.66	1.23	6.29	0.02
AHP-450 °C	1.59	0.07	0.26	1.61	3.86	1.11	2.31	0.13	11.09	1.04	0.09	0.78	4.80	0.37	25.39	0.03

### Organic petrography

**Figure 5:**
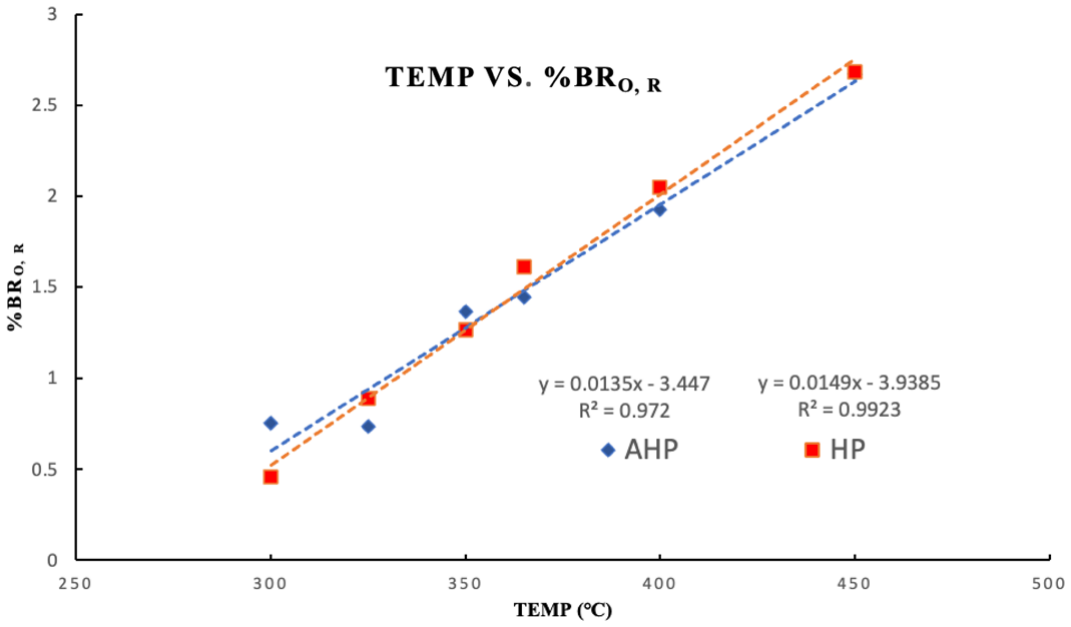
Cross-plot showing the bitumen random reflectance values trend (%Bro, r) with increasing temperature in anhydrous pyrolysis (AHP) and hydrous pyrolysis (HP).

Organic petrography demonstrates optical and structural evolution in macerals which varied for each maceral. Due to the scarcity of vitrinite particles, and to be consistent with measurements throughout the entire experiments, bitumen reflectance (%Bro, r) rather than vitrinite was examined. Results can be found in Table 6. Bitumen reflectance values (%Bro, r) increased with temperature during both the AHP and HP tests, but the trend was sharper in the presence of water (Fig. 5).

**Table 6:** Mean bitumen reflectance (%Bro, r) values under HP and AHP conditions.

Condition	Original	300 °C	325 °C	350 °C	365 °C	400 °C	450 °C
%Bro–HP	0.54	0.45	0.89	1.26	1.61	2.04	2.68
%Bro–AHP	0.54	0.75	0.73	1.36	1.44	1.92	2.67

## Discussion

### The role of water in impacting Rock–Eval parameters

In general, the organic matter content of source rocks can deteriorate dramatically during geologic time because of greater burial depth and temperature. Hydrocarbon production and expulsion are two major mechanisms that contribute to the depletion of OM^26^. This means that as the temperature increased in the AHP and HP experiments, a greater amount of organic matter was consumed during pyrolysis, resulting in a greater volume of hydrocarbons produced. Similarly, Valentine et al.^27^ demonstrated that the TOC content decreased as thermal maturity advances in the New Albany Shale (containing marine Type II kerogen) under HP conditions. Further, it has been shown that, as thermal maturity increases, catagenesis leads to the cleavage of aliphatic components of solid bitumen, resulting in a considerable decrease in TOC^28–30^.

As was reported in the Results section, variations in terms of both decreasing and increasing trends for HI were observed. It is important to note that an increase in the HI was also observed by Lohr and Hackley^31^ who reported an increase in the HI values for the Wilcox and Freestone coals (terrigenous Type III kerogen) when the original OM was heated to 300 °C, followed by a decrease in the HI values when the original OM was the subject of HP. This increase in HI with temperature was attributed to sample heterogeneity^31^. It should be emphasized that the HI value of the original (unheated) sample decreased less under AHP conditions than under HP conditions, a trend also observed by Moyer and Prasad^32^. This signifies that the sample retained a larger generation potential under AHP conditions than under HP conditions. Considerably more TOC is lost when there is no water in the system, but the residual TOC has higher hydrocarbon potential than the residual TOC in the hydrous test chamber.

Except for the extracted OM during the AHP test, T_max_ values increased with temperature under both HP and AHP settings, suggesting that thermal maturity advanced. Similarly, Huizinga et al.^33^ reported a regular rise in the T_max_ values of the Miocene Monterey Formation (kerogen Type II) during HP simulation. Additionally, Birdwell et al.^34^ observed an increasing trend in the T_max_ values for the OM from the Boquillas Shale (containing Type II kerogen) when the sample was heated from 300 °C to 330 °C.

Neither the HP nor the AHP tests demonstrate a consistent increase in T_max_ values with increasing temperature. This is important when they are compared with the measured %Bro, r values (Table 6). The latter exhibited a steady increase in both the HP and AHP scenarios. Thus, %Bro, r can be a better proxy for thermal maturity than T_max_. This was also concluded by Lohr and Hackley^31^ whereby organic petrography was found to be a better and more consistent approach for maturity assessment compared to the commonly performed programmed pyrolysis. This is because T_max_ cannot be measured accurately at high maturity because the S_2_ peak is very small and/or wide^12,35^.

**Figure 6:**
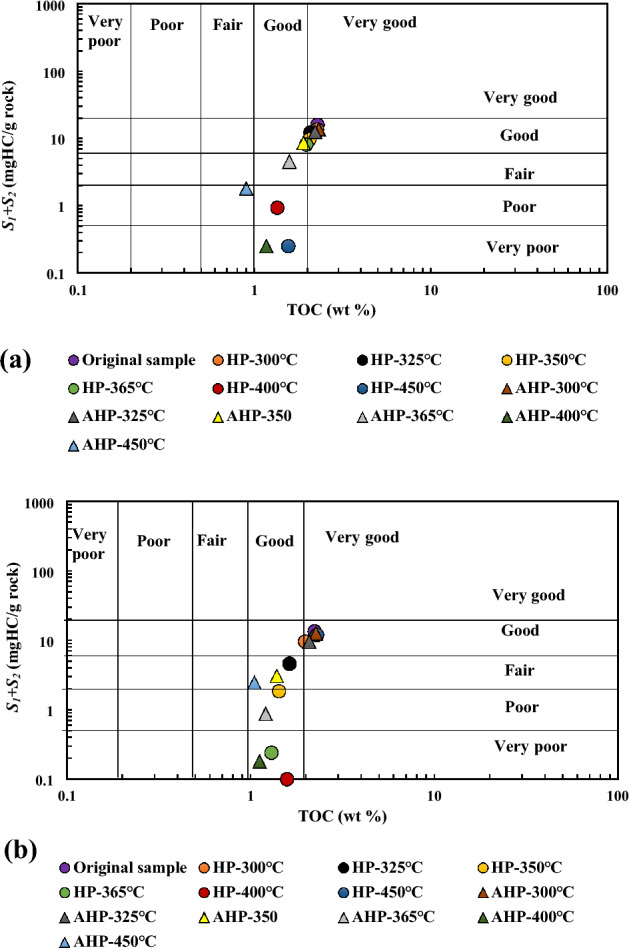
Change in hydrocarbon generation potential with increasing temperature during AHP and HP tests for un-extracted (**a**) and extracted OM (**b**).

Furthermore, a cross plot of TOC vs. S_1_ + S_2_ was used to investigate the variations in hydrocarbon generation potential of the un-extracted (Fig. 6a) and extracted (Fig. 6b) OM. Both un-extracted and extracted samples have a high potential for hydrocarbon generation. It is also evident that the hydrocarbon generation potential of both un-extracted and extracted samples decreased as thermal maturity increased during both AHP and HP experiments.

A general overview of all parameters, TOC, S_2,_ and S_1_ + S_2_, showed that the OM ability to generate hydrocarbons in the absence of water was inferior compared to hydrous pyrolysis where water is present in the system. This is in agreement with Lewan^7^ who stated that AHP reduces hydrocarbon production in comparison to HP. Furthermore, the production index (S_1_/(S_1_ + S_2_)) for both un-and extracted OM implied an improvement under both AHP and HP conditions, with the trend being more pronounced when water exists in the system. Indeed, the production index reveals that oil was generated in HP under lower thermal stress levels than under AHP. Similarly, Birdwell et al.^34^ found that the production index increased when OM from the Boquillas Shale was heated to temperatures between 300 and 330 °C under HP conditions.

It is worth mentioning that some RE-derived parameters (e.g., TOC, HI, and S_2_) do not show a systematic change as is expected during pyrolysis. This could originate from sample heterogeneity (mixed kerogen types), which affects kerogen kinetics and activation energy, thus the properties of produced hydrocarbons following each step of pyrolysis^36^.

### Gas chromatography (GC) and isoprenoid changes under AHP and HP conditions

It should be noted that many light hydrocarbon components were not measured during the quantitative gravimetric procedure, particularly over the temperature range of 400–450 °C (the incremental step of huge light hydrocarbon generation). Furthermore, GC as well as GC–MS analyses were not able to detect such light compounds. This explains why unanticipated quantities of n-alkanes were detected as the temperature increased, particularly beyond 400 °C (Tables 2 and 3).

**Figure 7:**
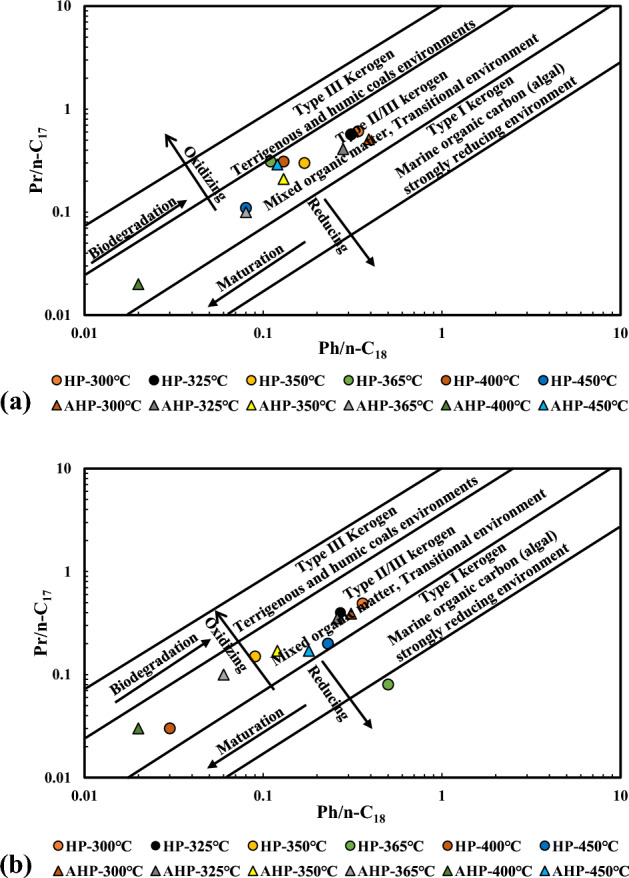
Plot of Pr/nC_17_ vs. Ph/nC_18_ demonstrating variations in normal alkanes and isoprenoids with increasing temperature during AHP and HP tests for (**a**) expelled byproduct and (**b**) residual byproduct. Redrawn from Peters et al.^40^.

Furthermore, unresolved complex mixtures (UCM) in some chromatograms (see Supplementary Data Figs. A and B) may lead to an inaccurate detection of normal alkanes and organic compounds. Indeed, co-elution of interferences from the sample matrices makes it difficult for GC and GCMS instruments to distinguish between interference compounds and target compounds^37^. Furthermore, the examined Qingshankou Formation contains mixed kerogen types (Fig. 7), which could lead to an amalgamation that would make it challenging for a routine GC approach to distinguish between their sources^38^. In the plot of Pr/nC_17_ vs. Ph/nC_18_ in both the expelled and residual byproducts (Figs. 7a and b), when temperature increased, the ratios of Pr/nC_17_ and Ph/nC_18_ were reduced overall under both HP and AHP conditions. These ratios are particularly sensitive to variations in temperature and the thermal maturation of the organic matter^39^. However, the residual byproduct exhibited a higher maturity than the expelled byproduct vs. temperature.

### Biomarker changes under AHP and HP conditions and the influence of water

In the expelled byproducts, it was found that the C_29_Ts increased when the temperature increased conditions (Table 4). The tricyclic hopane is used as a thermal maturity marker^40,41^. An increase in its value with HP was expected since hopanes are expelled from the kerogen at higher maturity levels. Thus, our observation from the expelled byproduct confirms the reliability of the C_29_Ts as a thermal maturity indicator. Conversely, the C_29_Ts values of the residual byproduct did not follow a systematic ascent under both HP and AHP conditions when temperature increased; for instance, between 300 °C and 350 °C, it decreased (Table 4).

The T_s_/T_m_ ratio can be considered as a biomarker which is a display of both lithology and thermal-maturity^40^. Considering the expelled byproduct during HP, this ratio steadily decreased from 0.83 to 0.53 when the sample was heated from 300 °C to 365 °C. Subsequently, the ratio increased from 0.53 to 0.73 when the temperature reached 450 °C. This means that between 365 °C and 400 °C the sample implied a clay-poor lithology since the T_s_/T_m_ ratio reached a value lower than 0.6^40^ while at other temperatures the formation acted as a clay-rich formation. However, during AHP, the T_s_/T_m_ ratio denoted a clay-rich lithology, although the ratio showed a decreasing trend between 300 °C and 350 °C (Table 4).

**Figure 8:**
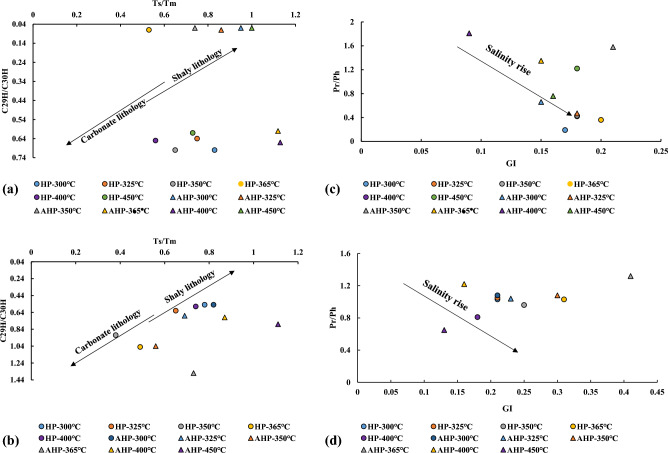
Plot of Ts/Tm vs. C_29_H/C_30H_ for expelled (**a**) and residual (**b**) byproducts showing the lithology; and plot of GI vs. Pr/Ph for expelled (**c**) and residual (**d**) byproducts showing salinity conditions. Redrawn from Peters et al.^40^.

Considering the residual byproduct, the T_s_/T_m_ ratio showed a decreasing trend between 300 °C and 350 °C during both the HP and AHP tests. Moreover, the residual product indicated both shale and carbonate lithology for the Qingshankou Formation sample, as was the case of the expelled byproduct (Table 5). In the expelled byproduct, the ratio of C_29_H/C_30_H fluctuated under HP and AHP conditions (Table 4). On the other hand, the C_29_H/C_30_H ratio for the residual byproduct increased progressively between 300 °C and 365 °C under both HP and AHP. The cross plot of T_s_/T_m_ vs. C_29_H/C_30_H for expelled and residual byproducts (Fig. 8a and b) generally represents a shaly lithology for the Qingshankou Formation.

Based on the vitrinite reflectance-equivalent values calculated from bitumen reflectance and presented in Table 6, the C_29_H/C_30_H ratio showed an increase during the oil window (defined to be from 300 to 350 °C in the above table). Nonetheless, at thermal maturities beyond the oil window (> 365 °C), this biomarker ratio declined. Similarly, Kotarba et al.^38^ reported an increasing trend of the C_29_H/C_30_H ratio under HP conditions when the temperature increased from 330 °C to 360 °C. Moreover, Liang et al.^42^ stated that the C_29_H/C_30_H ratio can be a reliable maturity indicator for coals and shales. This ratio is affected by hopane demethylation^43,44^ since the C_29_ hopane is thermally more stable than the C_30_ hopane. The C_29_H/C_30_H ratio revealed an expected increasing trend within the oil window. Overall, these outcomes suggest that C_29_H/C_30_H ratio can be a valid thermal maturity measure only within the oil window, but not beyond it (Table 6).

Gammacerane Index (GI) values have a wide range, from 0.09 to 0.20 in the expelled products and from 0.13–0.41 in the residual products (Fig. 8c,d). However, GI = 10 × gammacerane/(gammacerane + hopane) values are not high enough to suggest elevated water salinity during deposition of the source rock. The GI of both expelled and residual byproducts did not change significantly during both the HP and AHP tests, although in the expelled byproduct this index increased between 300 °C and 365 °C under HP (Table 4). An increase in GI within this range was also seen for the residual byproduct under both HP and AHP conditions (Table 5). The Pr/Ph ratio of marine organic matter is < 2, of lacustrine OM is in the range of 1–3, and is < 1.0 in a hypersaline environment^40^. In our study, Pr/Ph in the expelled products ranges from 0.36 to 1.81 and in the residual products from 0.18 to 1.32. Seven ratios in the expelled products are < 1.0 and four in the residual products are < 1.0 (Tables 4 and 5). The above ranges fall within the lacustrine and marine OM, with several falling in the hypersaline range. The Pr/Ph vs. GI plots (Fig. 8c,d) confirms that the mainly lacustrine depositional environment of the Qingshankou Formation was periodically inundated by saline marine waters (probably by marine transgressions), which suggests there was a communication between the two environments. However, the OM in the lake did not attain high enough GI to indicate that density stratification in the water column with depth occurred.

Regarding the C_23_ tricyclic terpane/C_24_ tetracyclic terpane ratio, an irregular variation in the expelled (Table 4) and residual byproducts (Table 5) with temperature increase was seen under both HP and AHP conditions. This ratio is commonly accepted as a depositional environment-related marker, and values greater than 1.0 reflect a marine depositional environment^45,46^. Thus, high values of this biomarker ratio regardless of variations with temperature could imply a marine depositional environment for the Qingshankou Formation. However, the Qingshankou Formation was deposited in a relatively deep lake setting impacted by marine incursions during a time of global sea-level rise^20^. Thus, higher values of C_23_ tricyclic terpane/C_24_ tetracyclic terpane ratio could have originated from marine incursions. Previous studies have shown the presence of different kerogen types including I and II in Qingshankou samples. This confirms the contribution of organic matter from both lake and marine environments during the deposition of the Qingshankou Formation^23,15,47^.

Likewise, the C_23_/C_24_ tricyclic terpane ratio oscillated when temperature increased under both HP and AHP in the expelled and residual byproducts (Tables 4 and 5). Such an inconclusive trend may stem from chemical reactions at each maturation level. It has been suggested that tricyclic terpanes (C_19–30_) were possibly derived from the degradation of a regular C_30_ isoprenoid (tricyclohexaprenol) generated by prokaryote membranes^48^ and thermally produced by thermal cleavage^42^. The C_30_ hopane biomarker of the expelled byproduct did not follow a particular pattern under both HP and AHP conditions except for a consistent decreasing trend in the AHP between 300 °C and 350 °C (Table 4). A decreasing C_30_ hopane was also recognized in the residual byproduct (Table 3) in both HP and AHP. However, there was a constant decrease in C_30_ hopane during the entire temperature range under AHP (Table 5).

Next are the C_19_/C_23_ and C_20_/C_23_ tricyclic terpane ratios, which were affected by temperature. In the expelled byproduct, the above ratios did not show any pattern (Table 4). However, in the residual byproduct, this ratio increased between 300 °C and 365 °C and then decreased until the final temperature of 450 °C under both HP and AHP conditions (Table 5). The C_19_/C_23_ and C_20_/C_23_ tricyclic terpane ratios increased within the main oil window, and then declined in the gas generation window and beyond. A similar trend of the C_19_/C_23_ and C_20_/C_23_ tricyclic terpane ratios under HP conditions was reported by Kotarba et al.^14^. They proposed that this could originate from variations in the rate of generation of each chemical compound. Molecules in the numerators can be produced more easily than those in the denominators. Once the state of higher maturity (at a high temperature) is reached, the conditions necessary for the creation of components in the denominators are met, leading to an inverse trend.

The ratio of C_30_ rearranged hopane to C_30_ hopane did not present any notable change in the expelled and residual byproducts and under both HP and AHP conditions. However, this ratio experienced a small increase as temperature surpassed 365 °C under AHP conditions (Tables 4 and 5). The above biomarker ratio can be utilized as a reference for thermal maturity assessment. The ratio rises as maturity advances because of the improved stability of the rearranged hopanes in comparison with the non-rearranged hopanes^49^. The observed irregularity of this ratio could have resulted from other causes, including lithology (mineral assemblages) in addition to maturity. Thus, based on our observations under AHP for both expelled and residual byproducts, the C_30_ rearranged hopane/C_30_ hopane ratio can be used with confidence as a thermal maturity-related diagnostic measure for maturities only within the late oil and gas windows.

### Aromatic compounds and the role of water

**Figure 9:**
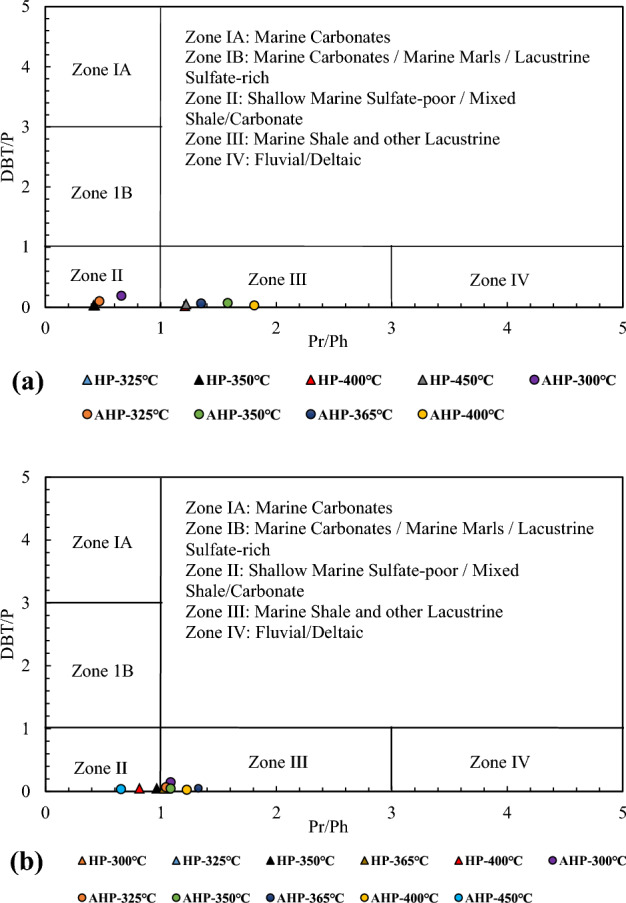
Plot of DBT vs. Pr/Ph ratio for the expelled (**a**) and residual (**b**) byproducts indicating the depositional environment. Redrawn from Peters et al.^40^.

Regarding the aromatic hydrocarbon fractions, the dibenzothiophene/phenanthrene (DBT/P) ratio did not vary significantly under HP conditions in both expelled (Table 4) and residual byproducts (Table 5). Nonetheless, a steady decrease in the above ratio was recognized under AHP conditions in both the expelled and residual byproducts. Likewise, Kotarba et al.^38^ reported a decline in DBT/P ratio values when the sample from the Lublin Coal Basin (Poland) was subjected to hydrous pyrolysis between 300 °C and 360 °C. Additionally, based on the DBT vs. Pr/Ph plot of the expelled (Fig. 9a) and residual byproducts (Fig. 9b), a contribution from both lacustrine and marine organic matter during the deposition of the Qingshankou Formation can be deduced.

The Methylphenantherene ratio (MPR = 2MP/1-MP) is widely used as a thermal maturity indicator. As seen in Table 4, there were fluctuations in the MPR values in the expelled byproducts under both HP and AHP. A general increase in the MPR values from the beginning to the end of the entire temperature interval (300–450 °C) for both HP and AHP in the residual byproduct was noticed (Table 5). Moreover, the MPR generally increased at higher temperatures. This suggests that aromatic compounds are more stable than biomarkers at higher maturities^50^. The concept of using the isomer distribution of methylphenanthrene as a maturity marker is dependent upon the thermal stability of isomers at various substituent locations. The isomers with *β*-substituents are more thermally stable than their counterparts with *α*-substituents. The 3- and 2-MP have a bridgehead position (*β*-type) and are believed to be more stable than the 9- and 1-MP (*α*-type), which can be produced via methylation from the rearrangement of 1- and 9-MP^51^. Furthermore, during pyrolysis, the methylphenantherene index (MPI = 1.5(2-MP + 3-MP)/(P + 1-MP + 9-MP)) did not show a fixed value. Akin to the MPR, the MPI displayed irregular variations with increasing temperature during HP and AHP. However, following the MPI, it is evident that the MPR increased at higher temperatures (350–450 °C) (Table 4). Considering the residual byproduct (Table 5), a general increase in the MPI values under both HP and AHP was inferred. This confirms the reliability of the MPI as a thermal maturity measure with the same concept as explained above for the MPR. In AHP pyrolysis, the heated sample generally showed higher MPI and MPR values than under HP, which was also reported by Lewan^7^.

Additionally, aromatic sulfur compounds have been a critical factor in determining the maturity of crude oils and source rocks^52–54^. Accordingly, a general increase in the methyldibenzothiophene ratio (MDR = 4-MDBT/1-MDBT) was apparent as temperature increased under HP and AHP for both expelled and residual byproducts (Tables 4 and 5). A slight variation in MDR values with increasing temperature suggests that this parameter can be a reliable thermal maturity index. Considering the methyldibenzothiophene (MDBT) isomers, 4-MDBT contains the *β-*substituent, whereas 1-MDBT bears an *α-*substituent. Therefore, MDR can be considered to be a good thermal maturity proxy on the same basis as the MPI^52^.

### Maceral changes and the role of water

**Figure 10:**
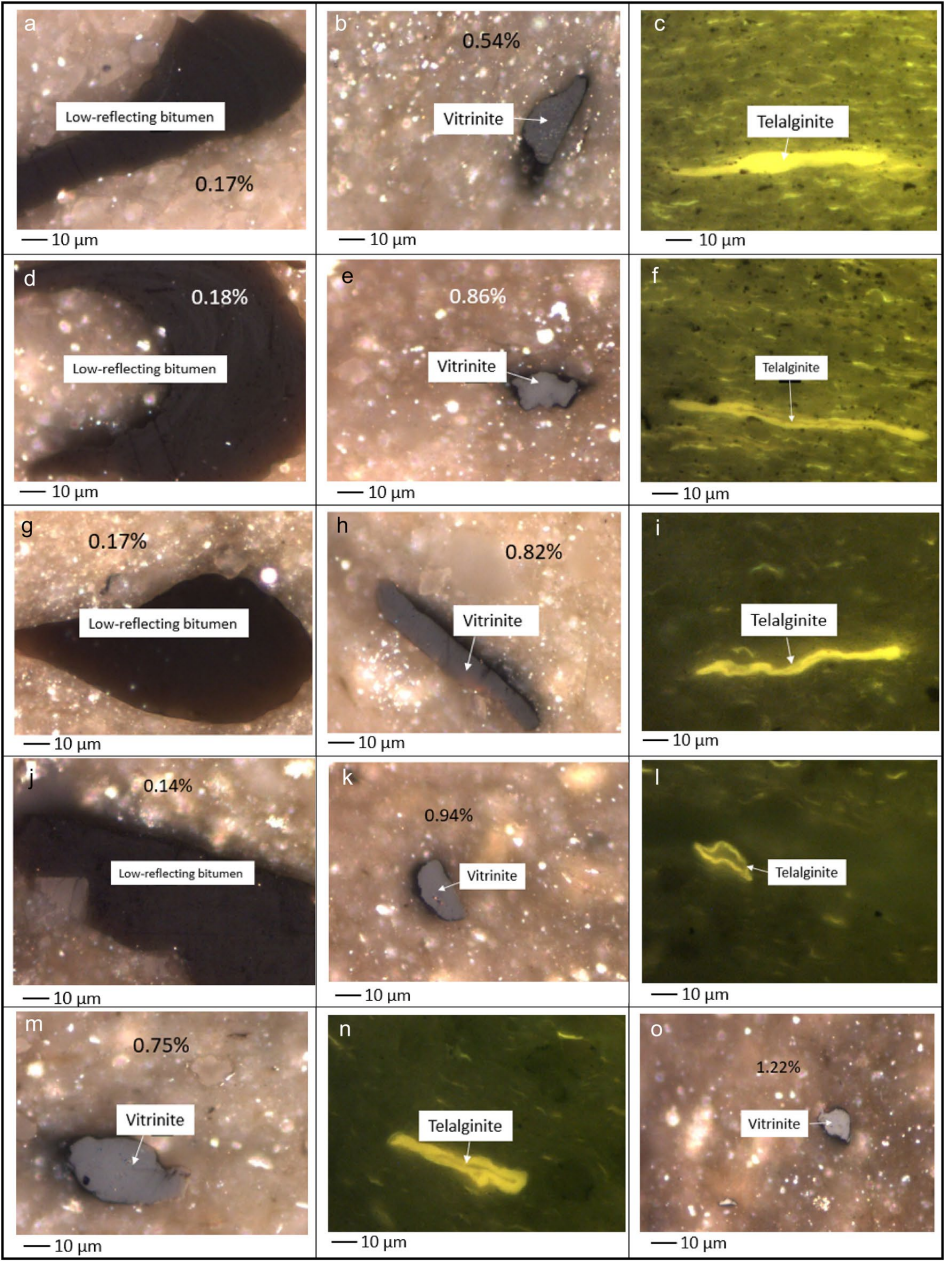

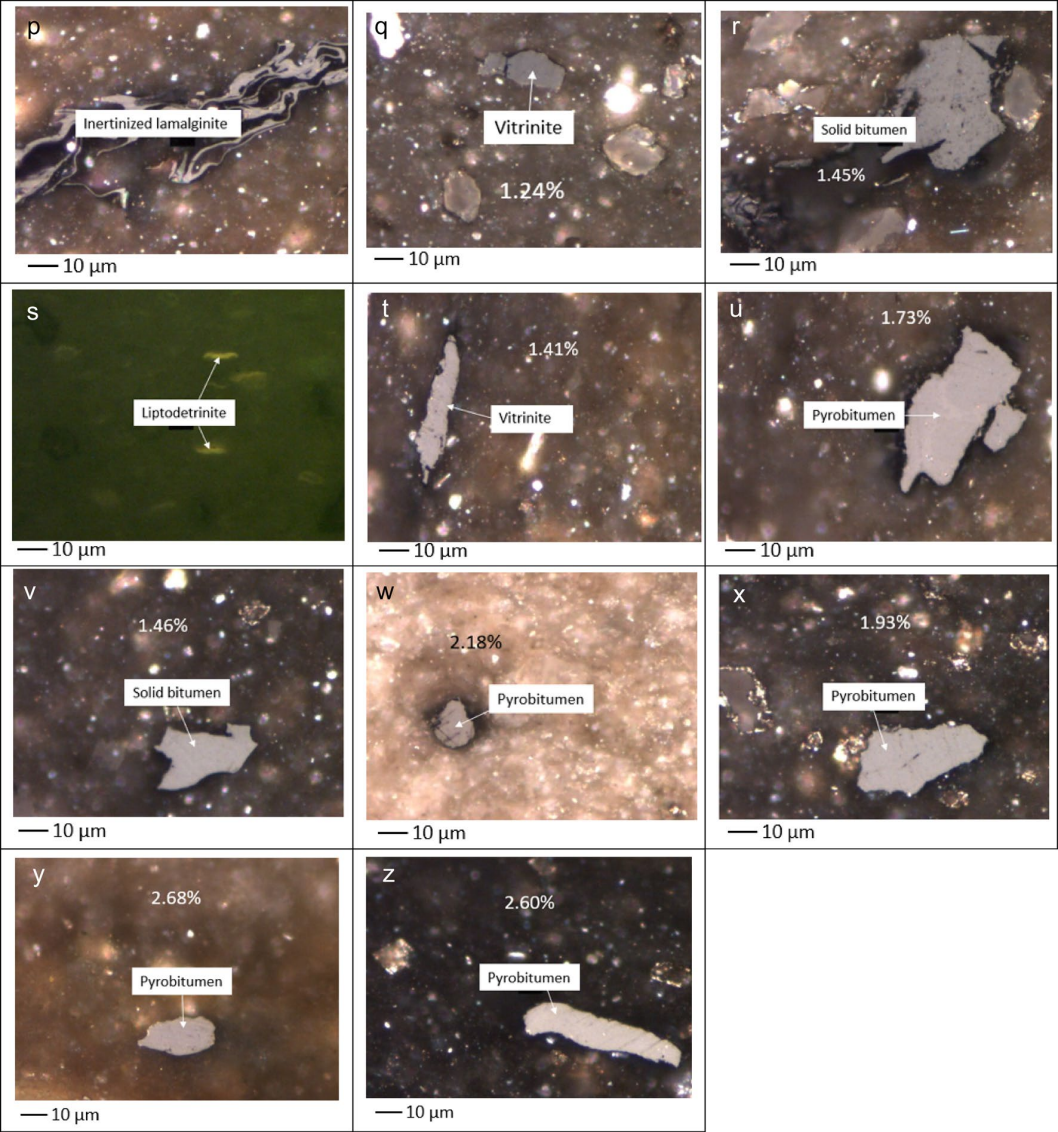
Photomicrographs of low-reflecting bitumen (**a**), vitrinite (**b**), and telalginite (**c**) in the original sample; low-reflecting bitumen (**d**), vitrinite (**e**), and telalginite (**f**) in the hydrous pyrolysis at 300 °C; low-reflecting bitumen (**g**), vitrinite (**h**), and telalginite (**i**) in the anhydrous pyrolysis at 300 °C; low-reflecting bitumen (**j**), vitrinite (**k**), and telalginite (**l**) in the hydrous pyrolysis at 325 °C; vitrinite (**m**), and telalginite (**n**) in the anhydrous pyrolysis at 325 °C; vitrinite (**o**), and inertinized telalginite (**p**) in the hydrous pyrolysis at 350 °C; vitrinite (**q**), solid bitumen (**r**), and liptodertinite (**s**) in the anhydrous pyrolysis at 350 °C; vitrinite-like (**t**) and pyrobitumen (**u**) in the hydrous pyrolysis at 365 °C; solid bitumen in the anhydrous pyrolysis at 365 °C (**v**); pyrobitumen under hydrous pyrolysis (**w**) and anhydrous pyrolysis (**x**) at 400 °C and pyrobitumen under hydrous pyrolysis (**y**) and anhydrous pyrolysis (**z**) at 450 °C. The length of the scale bar is $10\mu$ m.

The original (unheated) sample contained vitrinite, low-reflecting bitumen, and telalginite (Fig. 10a–c). When the sample was heated to 300 °C, alterations to the appearance of macerals occurred. The fluorescence intensity of the telalginite decreased when the temperature reached 300 °C under both HP and AHP conditions (Fig. 10f and i). However, such a decrease in the fluorescence intensity was more noticeable during HP. Moreover, an increase in vitrinite reflectance was observed when the original sample was heated to 300 °C under both HP and AHP (Fig. 10b,e,h). Yet, the vitrinite maceral experienced a higher increase in reflectance under HP (Fig. 10e) compared to AHP (Fig. 10h). It is worth mentioning that calculated vitrinite reflectance values for the sample under both HP and AHP conditions shows that heating at 300 °C is a sufficiently high temperature to artificially ‘mature’ the sample into the main oil generation window. Vitrinite, bitumen, and telalginite were observed at 300 °C under both HP and AHP conditions (Fig. 10d–i) while telalginite exhibited higher fluorescence intensity under AHP than under HP (Fig. 10f vs. i). This was reversed for vitrinite whereby a higher reflectance was measured under HP than under AHP (Fig. 10e vs. h).

At 325 °C, telalginite and vitrinite macerals distinguished under both HP and AHP (Fig. 10j–n). Akin to 300 °C, vitrinite particles displayed higher reflectance under HP (Fig. 10k) than under AHP (Fig. 10m). Likewise, telalginite particles still exhibited higher fluorescence intensity under AHP (Fig. 10n) than under HP (Fig. 10l). Next, at 350 °C, telalginite was absent under HP conditions and only a particle of inertinized laminated alginite could be seen (Fig. 10p). On the other hand, liptodertinite, as a member of the liptinite maceral group, was detected under AHP (Fig. 10s). Additionally, some vitrinite (Fig. 10o and q) and bitumen (Fig. 10r) particles were seen under AHP. Thus, it can be deduced that telalginite is not very resilient to temperature and disappears at lower temperatures under HP compared to AHP conditions. This means that water can become a catalyzer in the conversion of telalginite. It is has also been proposed that the loss of telalginite fluorescence is due to the conversion of this highly oil-prone maceral to liquid hydrocarbons during the oil generation window at lower temperatures^50^.

Organic petrography at 365 °C confirms that liptinite-group macerals cannot be found even under AHP. In this temperature, vitrinite-like (Fig. 10t) and pyrobitumen (Fig. 10u) particles were seen under HP while in the AHP pellet, bitumen was visible (Fig. 10v). Although bitumen particles were present in both AHP and HP, the difference in their reflectance allowed for their differentiation. The particle that existed under the AHP (Fig. 10v) had lower reflectance (%Bro, r = 1.46) and was identified as solid bitumen, whereas the one in the HP sample (Fig. 10u) had a higher reflectance (%Bro, r = 1.73) thus was pyrobitumen.

Furthermore, at 400 °C in the HP, the main components were vitrinite, pyrobitumen, and inertinized fragments. The pyrobitumen particle at this temperature (Fig. 10w) had %Ro of 2.18 (Fig. 10w) while the reflectance of pyrobitumen was 1.93% under AHP (Fig. 10x). Finally, at 450 °C, the remaining pyrobitumen particles in HP and AHP had different characteristics with the one under HP (Fig. 10y) having a slightly higher reflectance (2.68%) than the pyrobitumen under AHP (2.60%) (Fig. 10z).

Overall, higher vitrinite reflectance values were measured in the presence of water, while higher fluorescent intensity of telalginite was seen in the absence of water. Therefore, one could infer that vitrinite macerals have higher resilience to heat compared to telalginite macerals. Cracking of bonds is a common response of all materials and compounds to temperature. It has been suggested that two mechanisms occur during the pyrolysis in the presence of water. The first is the ability of water to solvate non-polar hydrocarbons at higher temperatures (> 300 °C), which is equivalent to organic solvents such as acetone, toluene, and benzene^55^. The second is the self-ionization of water, when an increase in temperature causes water to release ions such as H_3_O^+^ and OH^-^. These ions are highly reactive and establish a bond with the cracked bonds. This impedes the reconnection of cracked bonds and aids the creation of C–C bonds, which leads to the generation of high molecular weight compounds. Also, water acts as a source of hydrogen, and with increasing temperature these hydrogen molecules react with free radicals. This has been proposed as the mechanism for the process of self-ionization^55^.

Therefore, the presence of water during pyrolysis reinforces the thermal cracking of bonds and retards the cross-linking of C–C bonds^56,57^. This process generates higher hydrocarbon yields in HP compared to AHP^7^. This is corroborated by the higher free hydrocarbon (S_1_) peak of pyrolysis in HP compared to AHP obtained in this study. Similarly, the notable changes in telalginite macerals at higher temperatures, such as the decrease of their fluorescent intensity and especially the loss of visible fluorescence at higher temperatures, might be due to their dissolving with water or the self-ionization mechanism of water promoting thermal cracking. This can generally signify the low resistivity of liptinite macerals to heating in the presence of water.

Previous studies have suggested that liptinite group macerals, such as alginite, have significantly more distinct and noticeable aliphatic stretching bands and substantially lower aromatic carbon absorption compared to vitrinite. Macerals of the huminite group (precursor of vitrinite) have a stronger aromaticity than those of the liptinite group^58,59^. Indeed, previous studies conducted via advanced methods such as Raman spectrometry indicated that aromaticity decreases with inertinite > vitrinite > solid bitumen > liptinite. It has been suggested that liptinite group macerals experience relatively higher compositional modification during thermal maturity compared to other macerals^34,60^. Liptinite group macerals are aliphatic as opposed to the aromatic nature of huminite group macerals^61^. Considering that the aliphatic bonds have lower resistance than aromatic ones, alginite macerals have a lower resistivity than vitrinite macerals. This could explain the decrease in fluorescence intensity and the disappearance of alginite macerals as pyrolysis temperature increased. It should also be noted that alginite has lower aromaticity than cutinite and sporinite within the liptinite group of macerals.

## Conclusions

Anhydrous pyrolysis and hydrous pyrolysis (AHP/HP) tests were carried out on expelled and residual byproducts from an immature sample from the Qingshankou Formation, Songliao Basin, China. The following conclusions can be drawn:

- Gas chromatography-mass spectrometry (GCMS) analysis showed both an increase and a decrease in Rock–Eval pyrolysis parameters and in biomarkers with increasing temperature during pyrolysis and for both the expelled and remaining hydrocarbon byproducts. The lack of a systematic change in RE parameters is attributed to sample heterogeneity (mixed kerogen composition) and its impact on kerogen kinetics. On the other hand, variations in aromatic compounds were negligible.
- The biomarker C_29_Ts increased with temperature during HP and AHP for the expelled byproduct and vice versa for the residual byproduct. Moreover, temperature caused the Ts/Tm ratio to first show an increase followed by a decrease during both pyrolysis settings, and for both the expelled and residual byproducts.
- Although the C_29_H/C_30_H ratio for the expelled byproduct fluctuated when the temperature increased during HP and AHP, the above ratio for the residual byproduct increased within the oil window as the temperature increased under both conditions.
- Neither the Gammacerane Index (GI) nor the C_30_ rearranged hopane to C_30_ hopane ratio changed considerably from their initial values while both the C_23_ tricyclic terpane/C_24_ tetracyclic terpane ratio and the C_23_/C_24_ tricyclic terpane ratio showed an increase and a decrease under both HP and AHP conditions for the expelled and the residual byproducts.
- Under both HP and AHP conditions, the C_19_/C_23_ and C_20_/C_23_ tricyclic terpane ratios within the oil window increased and then decreased as maturity progressed.
- Under AHP, a gradual decrease in the DBT/P ratio was recorded for both expelled and residual byproducts. However, an overall increase in the methylphenantherene ratio (MPR) and methylphenantherene index (MPI) for the residual byproduct was observed regardless of the pyrolysis pathway.
- An increase in the methyldibenzothiophene ratio (MDR) was detected for both expelled and residual byproducts.
- Bitumen reflectance (%Bro, r) increased and optical and structural changes occurred in macerals with temperature, implying the substantial role water played in modifying maceral characteristics (reflectance and fluorescence) during the generation and expulsion of oil either by solvating non-polar hydrocarbons at higher temperatures or through the process of self-ionization.
- This study provides valuable insights into the oil-oil and oil-source correlations within the examined formation. By analyzing the chemical variations of this source rock, which has a unique biogenic origin in comparison to other source rocks worldwide, it contributes to an enhanced understanding of the role of water in petroleum production and organic matter (OM) evolution. These findings have the potential to contribute to the development of an updated model that captures the complexities of OM evolution and its relationship to the presence of water in petroleum production in the field.

## Supplementary Information

Supplementary Figures.

## Data Availability

The datasets used and/or analyzed during the current study available from the corresponding author on reasonable request.
