# Supplementary material for: The role of water in the laboratory thermal advancement of immature type I kerogen from the Cretaceous Qingshankou Formation in China
Source: Sci Rep. 2023 Jul 4;13:10815. doi: 10.1038/s41598-023-38013-z (PMC10319793; doi:10.1038/s41598-023-38013-z)
Supplement: Supplementary file 1 — Supplementary Figures. [file 41598_2023_38013_MOESM1_ESM.docx]

**Supplementary Material: GC-MS spectrum**


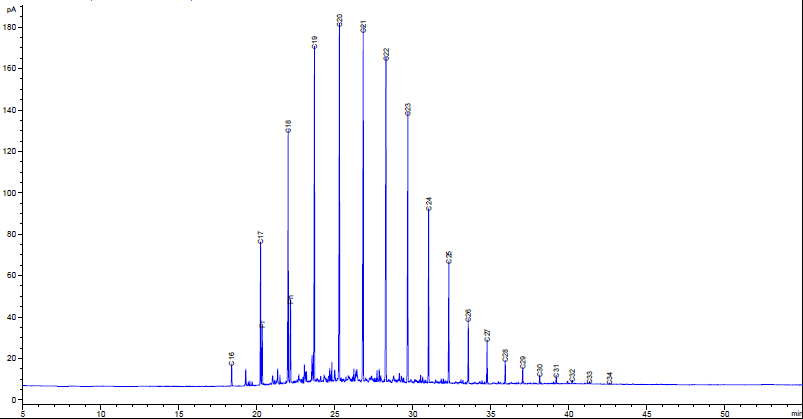


**(a)**


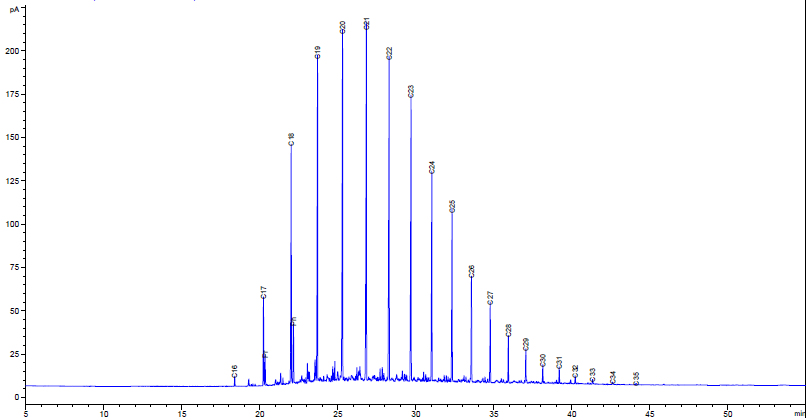


**(b)**


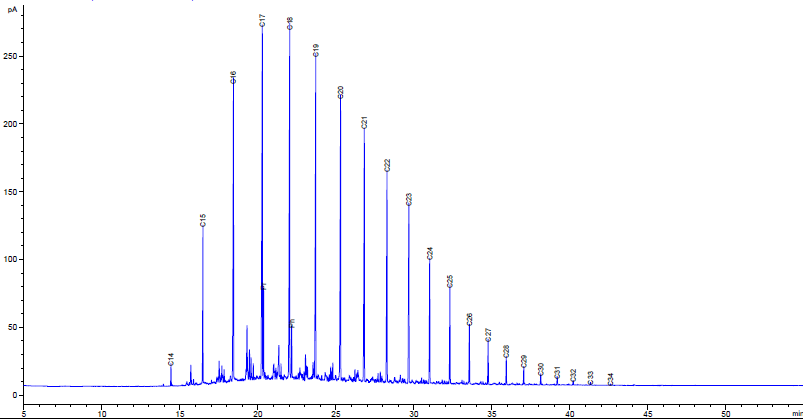


**(c)**


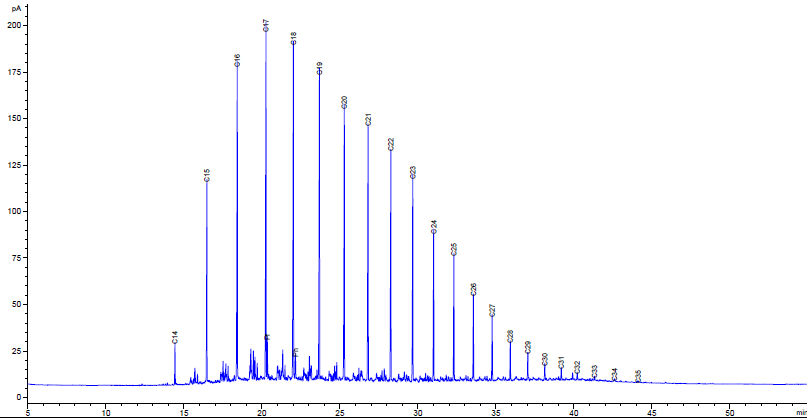


**(d)**


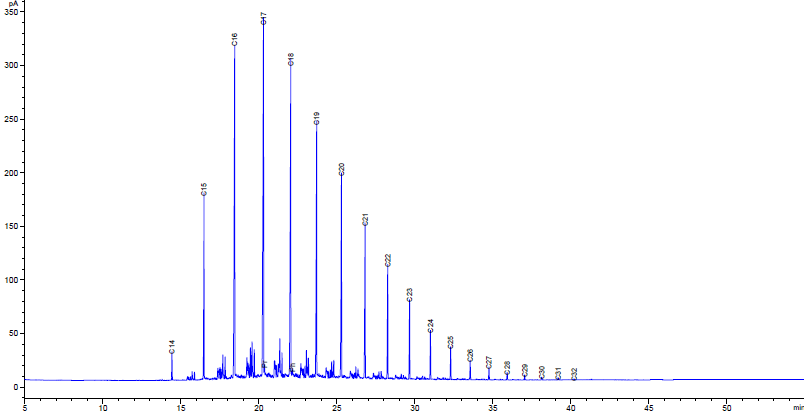


**(e)**


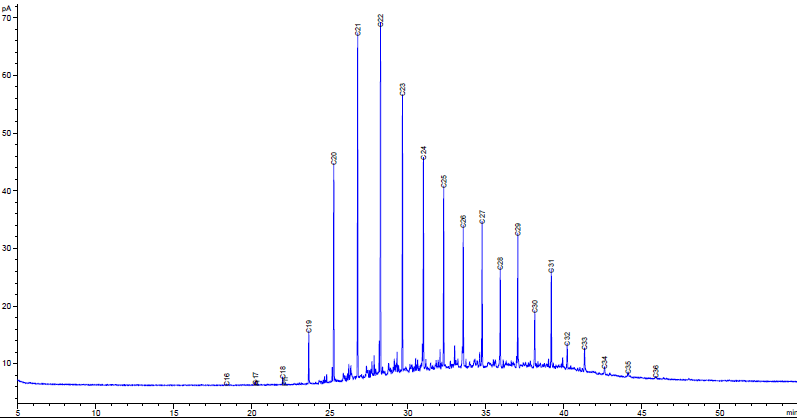


**(f)**


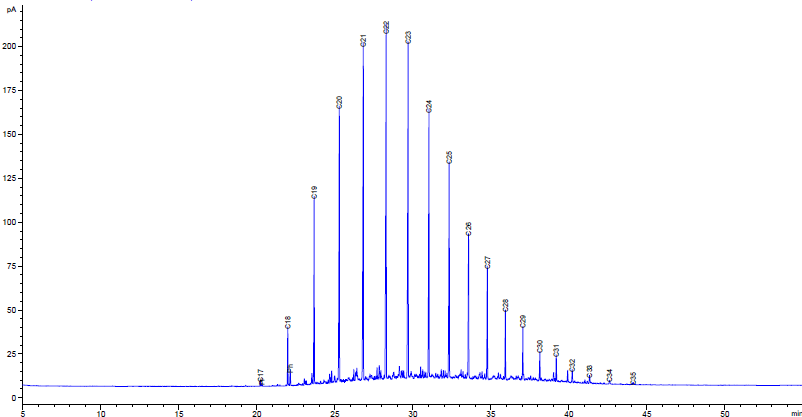


**(g)**


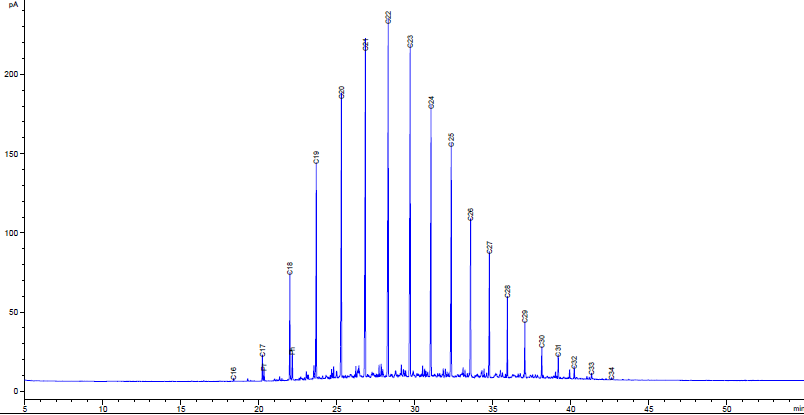


**(h)**


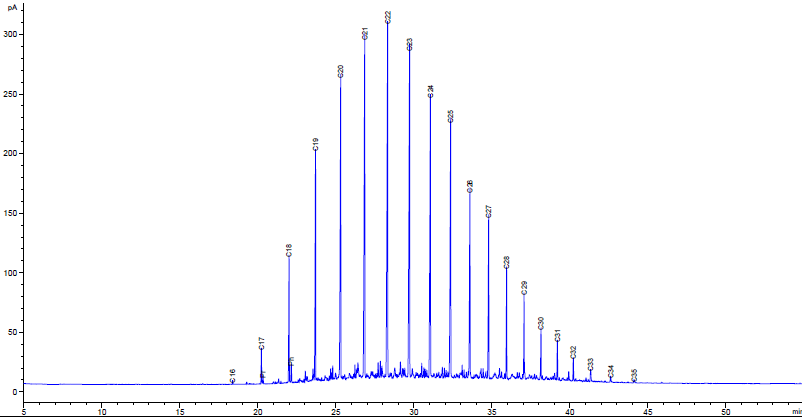


**(i)**


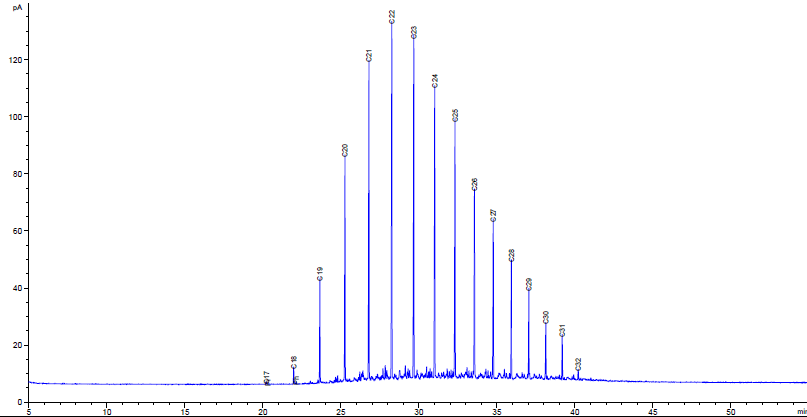


**(j)**


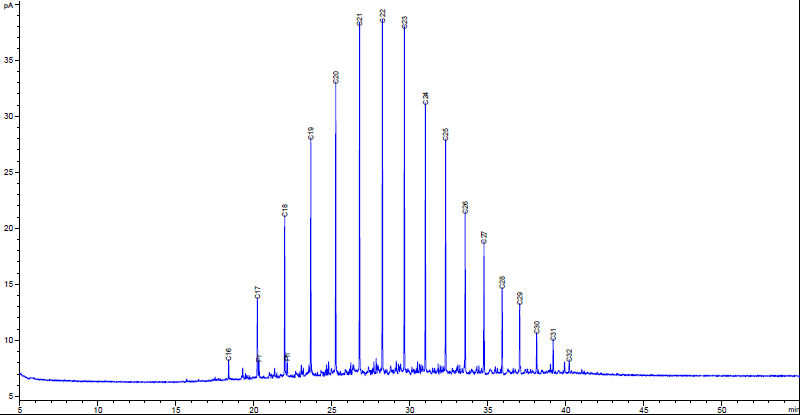


**(k)**


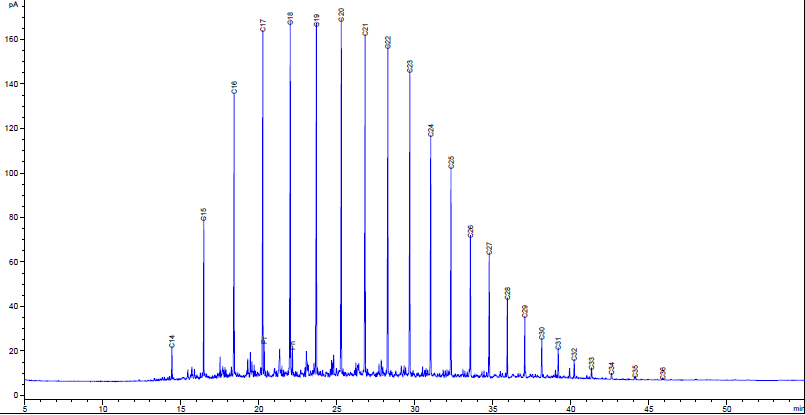


**(l)**

**Fig. A.** Gas chromatograms of the expelled byproduct under HP conditions at temperatures: 300℃ (a), 325℃ (b), 350℃ (c), 365℃ (d), 400℃ (e), and 450℃ (f) and under AHP conditions at: 300℃ (g), 325℃ (h), 350℃ (i), 365℃ (g), 400℃ (k), and 450℃ (l).


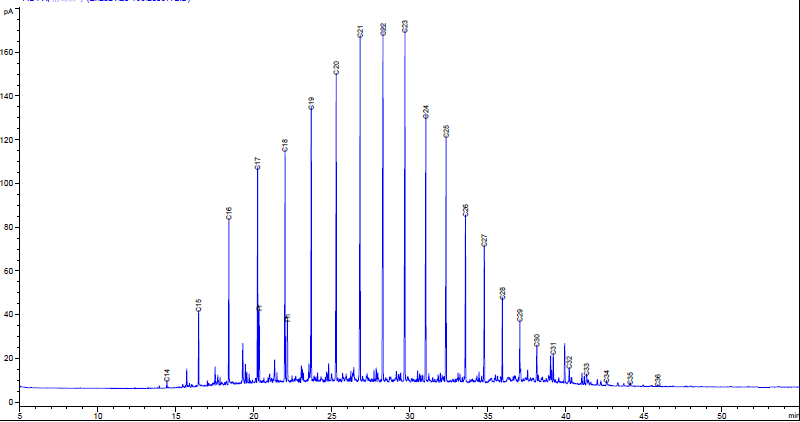


**(a)**


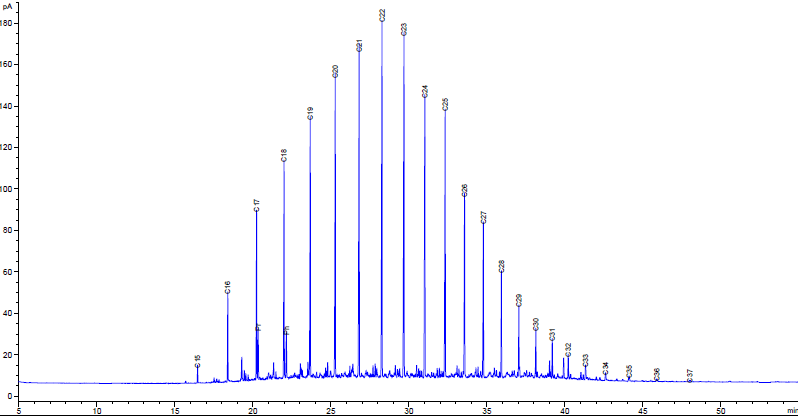


**(b)**


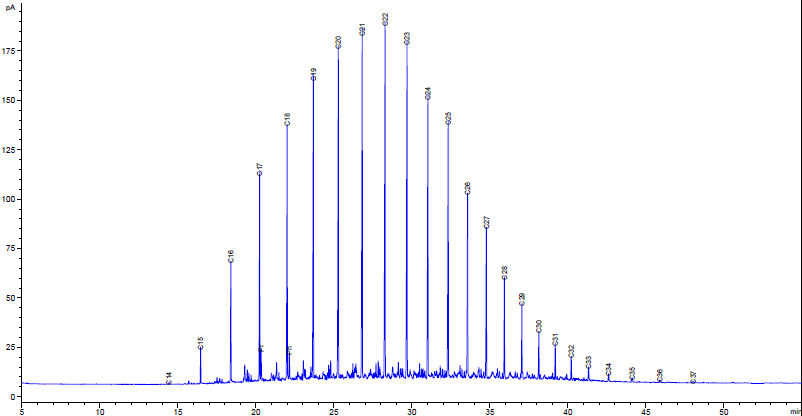


**(c)**


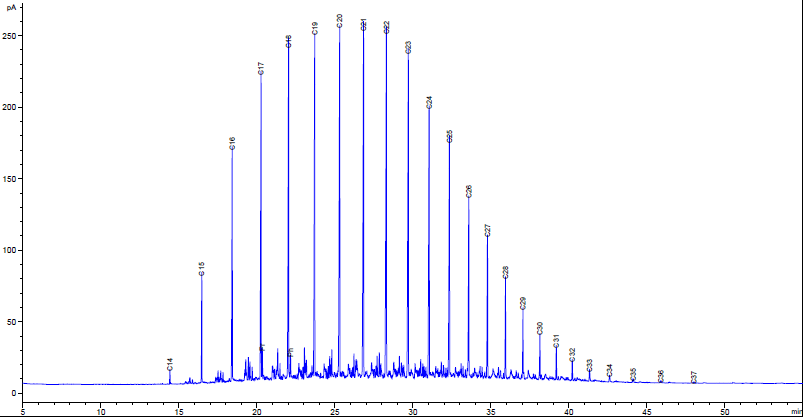


**(d)**


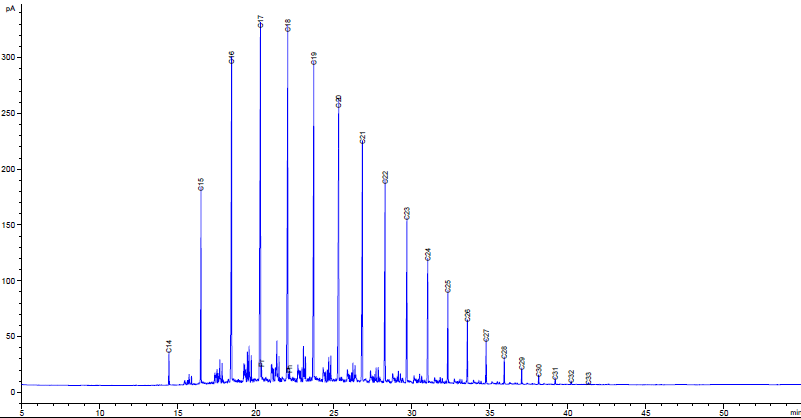


**(e)**


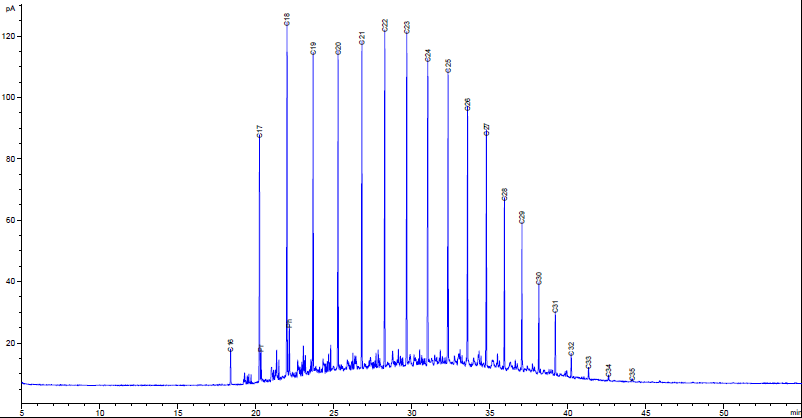


**(f)**


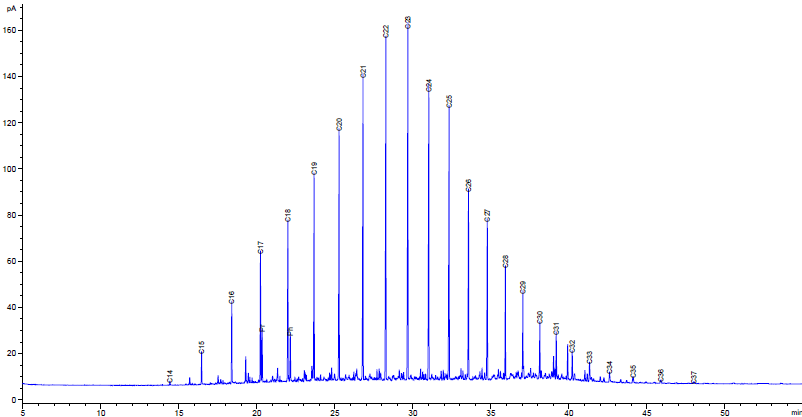


**(g)**


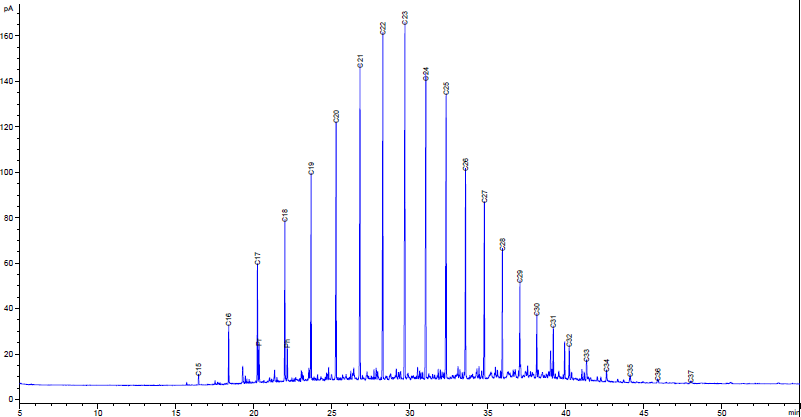


**(h)**


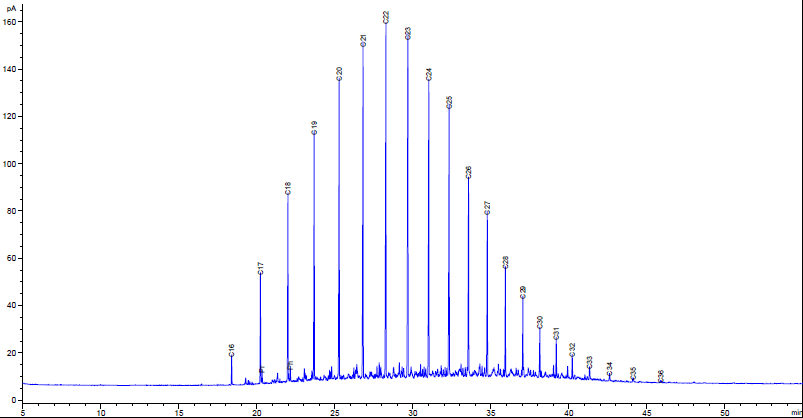


**(i)**


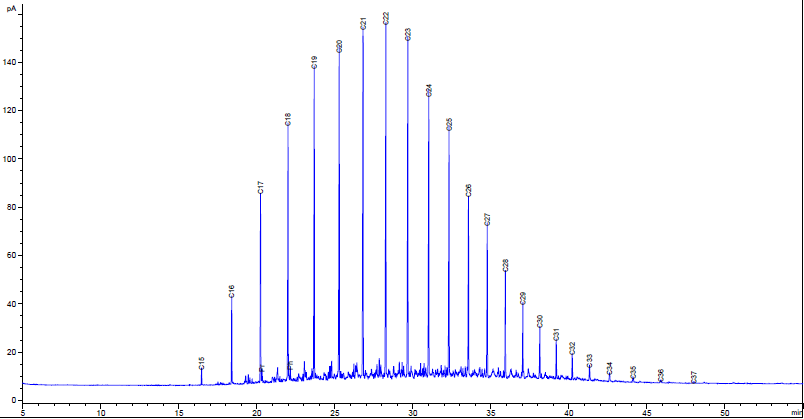


**(j)**


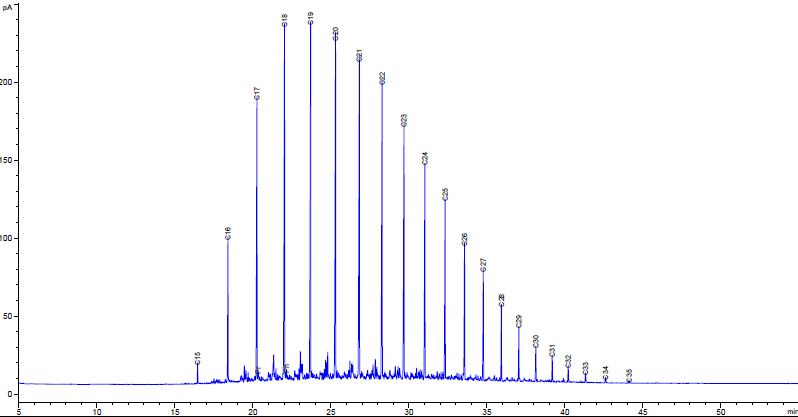


**(k)**


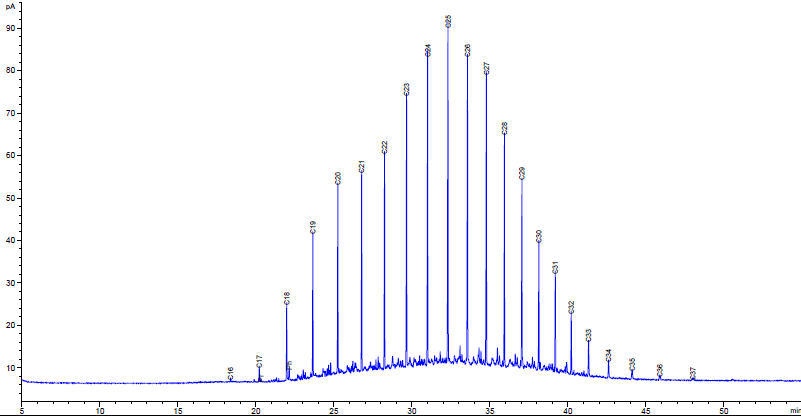


**(l)**

**Fig. B.** Gas chromatograms of the residual byproduct under HP conditions at temperatures: 300℃ (a), 325℃ (b), 350℃ (c), 365℃ (d), 400℃ (e), and 450℃ (f) and under AHP conditions at: 300℃ (g), 325℃ (h), 350℃ (i), 365℃ (g), 400℃ (k), and 450℃ (l).
